# Advances in the study of senescence mechanisms in the genus *Paeonia*

**DOI:** 10.1093/hr/uhae344

**Published:** 2024-12-06

**Authors:** Yuxuan Wang, Miao Sun, Wei Zhu, Le Chen, Shaocai Zhu, Jiageng Zhao, Jaime A Teixeira da Silva, Xiaonan Yu

**Affiliations:** School of Landscape Architecture, Beijing Forestry University, Beijing 100083, China; Beijing Key Laboratory of Ornamental Plants Germplasm Innovation & Molecular Breeding, National Engineering Research Center for Floriculture, and Laboratory of Urban and Rural Ecological Environment, Beijing 100083, China; School of Landscape Architecture, Beijing Forestry University, Beijing 100083, China; Beijing Key Laboratory of Ornamental Plants Germplasm Innovation & Molecular Breeding, National Engineering Research Center for Floriculture, and Laboratory of Urban and Rural Ecological Environment, Beijing 100083, China; Guangdong Key Laboratory of Ornamental Plant Germplasm Innovation and Utilization, Environmental Horticulture Research Institute, Guangdong Academy of Agricultural Sciences, Guangzhou 510640, China; School of Landscape Architecture, Beijing Forestry University, Beijing 100083, China; Beijing Key Laboratory of Ornamental Plants Germplasm Innovation & Molecular Breeding, National Engineering Research Center for Floriculture, and Laboratory of Urban and Rural Ecological Environment, Beijing 100083, China; School of Landscape Architecture, Beijing Forestry University, Beijing 100083, China; Beijing Key Laboratory of Ornamental Plants Germplasm Innovation & Molecular Breeding, National Engineering Research Center for Floriculture, and Laboratory of Urban and Rural Ecological Environment, Beijing 100083, China; School of Landscape Architecture, Beijing Forestry University, Beijing 100083, China; Beijing Key Laboratory of Ornamental Plants Germplasm Innovation & Molecular Breeding, National Engineering Research Center for Floriculture, and Laboratory of Urban and Rural Ecological Environment, Beijing 100083, China; Independent Researcher, Ikenobe 3011–2, Kagawa–ken 761–0799, Japan; School of Landscape Architecture, Beijing Forestry University, Beijing 100083, China; Beijing Key Laboratory of Ornamental Plants Germplasm Innovation & Molecular Breeding, National Engineering Research Center for Floriculture, and Laboratory of Urban and Rural Ecological Environment, Beijing 100083, China

## Abstract

Tree and herbaceous peony are considerably important ornamental plants within the genus *Paeonia*, and hold substantial horticultural value. This review summarizes the progress in research on the senescence mechanisms of tree and herbaceous peony flowers, focusing on the regulation of gene expression, hormonal interactions, and the influence of environmental factors on senescence. Using high-throughput sequencing technologies, key genes displaying differential expression during senescence have been identified, and these play central roles in hormone signaling and cellular senescence. The interactions among plant hormones, including ethylene, abscisic acid, gibberellins, cytokinins, and auxins, also play key roles in the regulation of senescence. Adjustments in antioxidant levels, as well as water and energy metabolism, are critical factors in the delay of senescence. Environmental factors, including light, temperature, drought, and salt stress, also significantly affect senescence. Additionally, this review proposes future research directions, including the expansion of the molecular regulatory network of senescence in *Paeonia*, the use of gene editing technologies like CRISPR/Cas9, multiomics studies, and exploratory comparative research on spatial biology senescence mechanisms. These studies aim to deepen our understanding of the molecular mechanisms that underlie senescence in *Paeonia* and provide a scientific basis for cultivar improvement and postharvest management of these ornamental commodities in the horticultural industry.

## Introduction

Tree peony (*Paeonia* sect. *Moutan*) and herbaceous peony (*Paeonia* sect. *Paeonia*) are both important ornamental plants within the family Paeoniaceae and genus *Paeonia*. Tree peonies are woody while herbaceous peonies are herbaceous, and both are highly popular horticultural commodities [1].

Tree peony, known as the ‘King of Flowers’ in China, plays a central role in garden landscaping and beautification [2]. Its elegant flowers and rich colors make it an indispensable commodity for garden aesthetics, park landscape design, and the cut flower market. In recent years, the cultivation of tree peony in Luoyang, China, has expanded significantly, with approximately 5136 hectares of cultivated area, and an annual production of 17 million stems, generating an economic benefit of 75 million RMB [3].

Herbaceous peony, known as the ‘Prime Minister of Flowers’ in China, also plays a key role in both garden landscaping and the cut flower market. In Heze, Shandong Province, in China, production reached 100 million stems in 2023, with a total sales value exceeding 400 million RMB [4]. Globally, herbaceous peony production has increased significantly over the past three decades, now covering over 25 countries, especially Europe, where production has increased about 50-fold [5]. This growth underscores the economic importance of herbaceous peonies worldwide.

Understanding the senescence of peony flowers is fundamentally important for extending their flowering period, enhancing their ornamental value, increasing market competitiveness, and expanding their economic value [4]. In the cut flower market, the ability to extend the postharvest vase life, which emerges from an understanding of the physiology of this process [6], is directly associated with higher profit margins. Additionally, research into the senescence mechanisms of peony flowers provides a theoretical basis for plant disease and pest management, enabling the development of effective strategies to enhance resistance, improve overall productivity, and promote the vitality of the peony cut flower industry [7].

Over the last decade, significant advances in plant senescence research have been achieved thanks to rapid developments in genomics and molecular biology. The application of high-throughput sequencing technologies has enabled researchers to identify genes and regulatory networks associated with senescence [8–10]. Furthermore, developments in gene-editing technologies, such as Clustered Regularly Interspaced Short Palindromic Repeats and CRISPR-associated protein 9 (CRISPR-Cas9), have allowed scientists to precisely modify specific genes to further investigate their roles in senescence [11–13]. In the realm of tree and herbaceous peony research, the application of these advanced technologies has begun to reveal genes controlling key biological processes such as flower longevity, petal senescence, leaf senescence, and flower opening [14–17]. These discoveries not only enhance our understanding of senescence in plants, but also provide new strategies for cultivar improvement and production management in horticultural practice.

This review synthesizes current scientific evidence from the perspectives of genomics, physiology, hormone regulation, and environmental science, and proposes future research directions. By adopting an interdisciplinary research perspective, we delve deeper into the molecular basis of the senescence mechanisms in tree and herbaceous peony and their relationship with environmental adaptability, providing a scientific foundation and innovative strategies for cultivar improvement, postharvest management, and sustainable development in the horticultural industry.

## Physiological and biochemical changes during senescence in *Paeonia*

### Cellular changes

Senescence is a critical stage in a plant’s life cycle and is characterized by profound cellular changes and complex biochemical processes. During this phase, plant cells undergo orderly degradation, including a decline in organelle function and restructuring of cellular components [18,19]. In the petals of tree and herbaceous peonies, cellular-level changes during senescence are a key area of research, with prominent features including programmed cell death (PCD), cell wall modifications, and autophagy.

#### PCD and cell wall changes

PCD plays a crucial role during the senescence of peony petals, beginning in the early flowering stages and becoming more pronounced during petal wilting. In the herbaceous peony *Paeonia lactiflora* ‘Zhong Sheng Fen’, scanning electron microscopy (SEM) and transmission electron microscopy (TEM) revealed key features of PCD, including vacuolar membrane rupture, organelle degradation (i.e. of mitochondria, the endoplasmic reticulum, and nucleus), and nuclear membrane rupture [20]. Similar processes were observed in tree peonies, such as in the upper shoots of *Paeonia* × *suffruticosa* ‘Luoyang Hong’, where insufficient lignin deposition and reduced cellulose content were linked to ‘withering’ or cell death, ultimately leading to a loss of tissue viability [21].

PCD, which is often accompanied by structural changes in the cell wall, contributes to visible symptoms of senescence. In *P. lactiflora* ‘Zhong Sheng Fen’, increased cellulase and pectinase activities were observed during senescence, facilitating the breakdown of cell wall components [20]. In tree peonies such as *Paeonia ostii* ‘Feng Dan’ and *P.* × *suffruticosa* ‘Luoyang Hong’, varying levels of lignin and cellulose accumulation in different shoot regions were correlated with their capacity to withstand seasonal senescence. Lignin accumulation in the lower parts of the shoot prevented ‘withering’, whereas the upper parts, characterized by lower lignin and cellulose levels, were more vulnerable to senescence and eventual PCD [21].

#### Membrane permeability and cell integrity

During the natural senescence of herbaceous peony flowers, such as *P. lactiflora* ‘Hong Feng’ and *P. lactiflora* ‘Yang Fei Chu Yu’, the selective permeability of cell membranes declined significantly, leading to increased solute leakage. The permeability increased by 84.8% from the color-displaying stage to the onset of senescence in *P. lactiflora* ‘Hong Feng’, contributing to the degradation of nucleic acids and proteins [22]. This increased permeability facilitates the uncontrolled movement of solutes, accelerating cellular senescence.

Exogenous treatments, such as 4% calcium chloride (CaCl_2_), can mitigate these changes by maintaining cell membrane integrity. In *P. lactiflora* ‘Yang Fei Chu Yu’ cut flowers, a 4% CaCl_2_ spray extended vase life by reducing membrane permeability [23]. Wang [24] demonstrated that a 4% calcium acetate (Ca(CH_3_COO)_2_) treatment combined with lateral bud removal in herbaceous peony cultivars like *P. lactiflora* ‘Chun Xiao’ reduced membrane permeability and increased structural components like cellulose and lignin, thereby enhancing stem strength and extending vase life, but the same treatment had a negative effect on *P. lactiflora* ‘Da Fu Gui’, indicating a cultivar-specific response to calcium treatment.

Melatonin (MT) treatment has also shown efficacy in maintaining cell membrane stability. In *P. lactiflora* ‘Hongyan Zhenghui’, treatment with 50 μmol·L^−1^ MT significantly enhanced stem strength by increasing lignin content and secondary cell wall thickness, thereby improving structural stability [25]. In *P. lactiflora* ‘Qi Hua Lu Shuang’ and *P. lactiflora* ‘Da Fu Gui’, Wang et al. [26] further confirmed that MT treatment at 50 μmol·L^−1^ significantly reduced electrical conductivity by up to 33.96%, supporting membrane integrity and maintaining postharvest flower quality. These treatments effectively delayed senescence by maintaining cellular integrity, highlighting their potential application in extending the vase life of peony flowers.

#### Lignification and the C/N ratio

The senescence process in peonies also involves changes in shoot structure, particularly in lignification and the carbon/nitrogen (C/N) ratio, which are crucial for shoot health and development. For instance, studies on the tree peony *P.* × *suffruticosa* ‘Luoyang Hong’ and *P. ostii* ‘Feng Dan’ showed that lower lignification in the upper internodes compared to the base was correlated with increased susceptibility to wilting. Besides lignification, the C/N ratio in the upper shoots was significantly lower than that at the base, indicating insufficient carbon assimilation, potentially contributing to increased susceptibility to wilting [21]. These findings suggest that variations in lignification and the C/N ratio are linked to the structural stability of shoots, potentially affecting their resistance to senescence-related wilting. These insights provide a foundation for developing targeted interventions to enhance lignification and carbon assimilation, potentially improving the resistance of peony shoots to senescence.

### Biochemical changes

Biochemical changes play a central role in senescence in plants, involving adjustments to antioxidant levels, water balance, and energy metabolism [27, 28]. These changes not only reflect the impact of individual biochemical events but also reveal complex interactions of various physiological responses that together determine the progression of senescence and longevity of flowers, thereby affecting their ornamental value and market competitiveness [29].

#### Effects of exogenous treatments on antioxidant mechanisms and oxidative stress in Paeonia cut flowers

To mitigate the oxidative stress associated with senescence, various exogenous treatments have been explored, focusing on enhancing antioxidant defenses. Senescence in *Paeonia* cut flowers is closely linked to oxidative stress, which can cause significant cellular damage if not effectively managed. During senescence, reactive oxygen species (ROS) such as hydrogen peroxide (H_2_O_2_) accumulate, leading to lipid peroxidation and cellular damage. The accumulation of malondialdehyde (MDA), a product of lipid peroxidation, serves as an indicator of oxidative stress levels [30].

Shi et al. [31] found that treatment with H_2_O_2_ accelerated the aging of *P. suffruticosa* ‘Luoyang Hong’ cut flowers, resulting in increased MDA levels and reduced vase life, indicating heightened oxidative stress. In contrast, ascorbic acid (ASA) treatment significantly decreased MDA levels by 6.7% and extended vase life by 0.8 days compared to untreated controls, demonstrating its protective effects against oxidative stress. This suggests that antioxidants like ASA can effectively mitigate ROS-induced damage and delay senescence in *Paeonia* cut flowers.

Further research explored other compounds that can modulate oxidative stress and improve flower longevity. MT enhanced antioxidant defense mechanisms in *P. lactiflora* cultivars. Wang et al. [26] demonstrated that treatment with at 50 μmol·L^−1^ MT significantly increased the activities of two key antioxidant enzymes, superoxide dismutase (SOD) and catalase (CAT), in *P. lactiflora* ‘Qi Hua Lu Shuang’ and *P. lactiflora* ‘Da Fu Gui’. This increase in antioxidant enzyme activity helped mitigate oxidative damage, ultimately extending the vase life of these cultivars. Similarly, silver nanoparticles (Ag-NPs) synthesized from *Eucommia ulmoides* leaf extract reduced oxidative stress markers and enhanced antioxidant enzyme activity in *P.* × *suffruticosa* ‘Luoyang Hong’ tree peony, extending vase life by 1.4 days [32].

Chlorine dioxide (ClO_2_) has also proven effective in managing oxidative stress in *P. suffruticosa*. Zhang et al. [33] noted that ClO_2_ treatment enhanced the activities of antioxidant enzymes like SOD, peroxidase (POD), and CAT, while reducing oxidative stress markers like MDA and H_2_O_2_, thereby extending the vase life of *P.* × *suffruticosa* ‘Luoyang Hong’ by 3.2 days compared to the control.

Silicon treatments have also been investigated for their ability to enhance antioxidant capacities in *Paeonia* cut flowers. Song et al. [34,35] found that silicon application increased the activities of SOD, CAT, and ascorbate peroxidase (APX), contributing to better management of oxidative stress. These treatments improved both flower quality and longevity, highlighting the role of silicon in enhancing oxidative stress response and extending vase life.

#### Effects of exogenous treatments on water balance in Paeonia cut flowers

In studies of senescence in tree and herbaceous peony, water metabolism plays a decisive role in maintaining the freshness of cut flowers and extending their vase life.

Zhao et al. [36] demonstrated that a 30 mg·L^−1^ NS treatment on *P. lactiflora* ‘Hong Yan Zheng Hui’ significantly enhanced water absorption and reduced microbial growth, increasing water uptake by 15%. Song et al. [34,35] demonstrated that treatment with 75 mg·L^−1^ silicon (Na_2_SiO_3_) enhanced water absorption in *P. lactiflora* ‘Euiseong’, leading to a significant increase in vase life by improving cell wall stiffness and porosity.

According to Ma et al. [32], Ag-NPs at concentrations of 5 and 10 mg·L^−1^ improved water retention in *P.* × *suffruticosa* ‘Luoyang Hong’ tree peonies, extending vase life by 1.2 days by optimizing water balance and enhancing flower diameter. Li et al. [37] found that by applying an 8% concentration of herbaceous peony polyphenols derived from the fermentation of peony flowers using yeast, like *Saccharomyces fibuligera* and *Saccharomyces cerevisiae*, increased vase life by 4 days and reduced water loss by 30% in *P. lactiflora* ‘Hong Yang Zheng Hui’. The fermentation process was crucial because it enhanced the content and bioactivity of the polyphenols, contributing to their effectiveness in extending flower longevity and reducing water loss. Similarly, ClO_2_ applied at 0.100 g·L^−1^ to *P.* × *suffruticosa* ‘Luoyang Hong’ increased water uptake by 37.5% and extended vase life by 3.2 days [33]. MT at 50 μmol·L^−1^ extended the stable water balance period significantly in *P. lactiflora* ‘Qi Hua Lu Shuang’ and *P. lactiflora* ‘Da Fu Gui’, highlighting its effectiveness in maintaining hydration levels [26]. Guo et al. [38] noted significant differences in water retention capabilities linked to petal thickness, with *Paeonia* intersubsectional hybrid ‘Haihuang’ (‘High Noon’) exhibiting better longevity due to its thicker mesophyll and epidermis, compared to *P.* × *suffruticosa* ‘Zhihong’.

These findings underline the critical role of tailored postharvest treatments in enhancing water metabolism, which is pivotal for prolonging the decorative life and freshness of peonies.

#### Changes in photosynthesis and respiration during Paeonia flower senescence

Photosynthesis and respiration are fundamental processes for the transformation of plant energy and growth [39,40]. During plant senescence, photosynthetic efficiency typically declines due to reduced chlorophyll content and decreased activity of photosynthesis-related enzymes, while respiration rates often increase to meet the energy requirements for cellular repair and defense responses [41, 42]. This inverse relationship significantly influences the energy balance during the aging process of *Paeonia* species.

A study on *P. ostii* ‘Feng Dan’ tree peony by Han et al. [43] found that optimal shading improved photosynthetic efficiency by increasing chlorophyll content and enhancing photosynthesis-related enzyme activities, which subsequently provided more energy for growth and delayed senescence. However, when shading was too intense, photosynthesis declined significantly, reducing energy availability and accelerating senescence. This highlights the importance of balancing shading intensity to maximize photosynthesis while minimizing the energy burden on respiration. Data on photosynthesis showed that light shading (18.4% reduction in PPFD) during early growth improved stomatal distribution and seed yield by 33.3%, emphasizing the role of photosynthesis in delaying senescence [44]. Moreover, Zhao et al. [45] showed that enhancing caffeoyl-CoA O-methyltransferase activity in *P. ostii* ‘Feng Dan’ improved drought tolerance and protected against oxidative damage by promoting lignin synthesis and ROS scavenging, thereby maintaining photosynthetic efficiency during senescence.

Additionally, Xie et al. [46] demonstrated that moderate shading (30% reduction in PPFD) in herbaceous peonies (*P. lactiflora* ‘Zi Feng Yu’ and *P. lactiflora* ‘Hong Feng Yu’) maintained chlorophyll levels, reduced photo-oxidative damage, and enhanced photosynthetic activity by up to 25% compared to unshaded plants. This increase in photosynthetic efficiency positively influenced respiration, reducing the need for energy-intensive repair processes and supporting metabolic activities necessary for delaying senescence.

During senescence, respiration plays a crucial role in managing the plant’s energy needs, particularly as photosynthesis declines. Shi et al. [42] found that in tree peony cultivars like *P.* × *suffruticosa* ‘Luoyang Hong’ and *P.* × *suffruticosa* ‘Hu Hong’, respiration rates surged by as much as 30% during the transition of flowers from blooming to senescence, coinciding with a significant drop in photosynthetic efficiency. The increase in respiration redistributed energy to support essential metabolic processes, such as cell maintenance and defense responses.

For herbaceous peony cut flowers, Wang et al. [47] observed that at full bloom in *P. lactiflora* ‘Tao Hua Fei Xue’, both respiration rates and ethylene (ETH) production peaked, while photosynthetic efficiency declined, marked by an 18% reduction in chlorophyll content. This metabolic imbalance accelerated senescence. However, the application of preservative solutions containing sucrose and salicylic acid moderated respiration rates by 15%, reducing the energy cost of accelerated senescence and extending the vase life of cut flowers.

Together, effective regulation of photosynthesis and respiration supports the energy-related physiological processes that are essential for enhancing the ornamental quality and extending the market value of peony flowers.

For a detailed overview of the efficacy and drawbacks of various preharvest and postharvest treatments on the vase life and physiological responses of cut flowers, refer to Tables 1 and 2.

**Table 1:** Enhanced vase life and physiological responses in *Paeonia* cut flowers: efficacy of preharvest and postharvest treatments

Treatment	Concentration	Control	Cultivar	Effect	References
ASA	0.01 M	Distilled water	*P.* × *suffruticosa* ‘Luoyang Hong’	Extended vase life by 0.8 days (20%), increased SOD by 8.1% at late vase-life stage, increased CAT by 15.3% (not significant) at late vase-life stage, decreased MDA by 4.1% (at 3 days).	[31]
Ag-NPs Ag-NPs	5 mg·L^−1^ 10 mg·L^−1^	Deionized water Deionized water	*P.* × *suffruticosa* ‘Luoyang Hong’ *P.* × *suffruticosa* ‘Luoyang Hong’	Extended the stage VI (onset of wilting) by approximately 0.3 days compared to the control group, extended optimal viewing period by 0.9 days. Increased flower diameter by 9.3%, MDA content was reduced by 20.5% to 16.1%.	[32] [32]
MT	50 μmol·L^−1^	Distilled water, 3% sucrose, and 200 mg·L^−1^ 8-HQ	*Paeonia lactiflora* ‘Qi Hua Lu Shuang’ *P. lactiflora* ‘Da Fu Gui’	SOD and CAT activities in ‘Qi Hua Lu Shuang’ and ‘Da Fu Gui’ increased by 22.47% and 33.96%, and 31.86% and 44.46%, respectively. Stable water balance extended by 6.0 days in ‘Qi Hua Lu Shuang’ and 5.7 days in ‘Da Fu Gui’.	[26]
ClO_2_	0.1 g·L^−1^	Distilled water	*P.* × *suffruticosa* ‘Luoyang Hong’	Extended vase life by 3.2 days, increased water uptake by 37.5%, enhanced SOD by 30.9%, POD by 31.5%, CAT by 10.0%, reduced MDA by 41.2%, and reduced H_2_O_2_ by 38.3%. Inhibited bacterial growth and decreased lignin content by 42.2%. Increased water uptake by 37.5%	[33]
preharvest CaCl_2_ spray	4%	Tap water	*P. lactiflora* ‘Yang Fei Chu Yu’	Extended vase life by 2.3 days (23%), increased flower diameter to 12.5 cm, and prolonged full bloom stage to 5 days.	[23]
NS	30 mg·L^−1^	Deionized water	*P.* × *suffruticosa* ‘Hong Yan Zheng Hui’	SOD up by 37.6%, CAT by 28.9%, MDA down by 21.7%, O_2_^−^ production rate reduced by 33.4%. Fresh weight gain peaked at 658 g on day 6, significantly higher than control. Increased relative water uptake by 15% and reduced microbial colonization, which was visually confirmed by scanning electron microscopy.	[36]
Polyamine inhibitors	0.1 mM D-Arg	Distilled water	*P. lactiflora* ‘Qi Hua Lu Shuang’	Extended vase life by 1.8 days, increased SOD activity by 7.8%, increased CAT activity by 17.3%, reduced MDA content by 5.6%.	[48]
Si Si + preservative (commercial floral preservative)	Preharvest: 0.75 g Na_2_SiO_3_ Postharvest: 75 mg·L^−1^ 75 mg·L^−1^ + preservative (glucose, citric acid; Si-free)	Distilled water Distilled water	*P. lactiflora* ‘Taebaek’ *P. lactiflora* ‘Taebaek’ *P. lactiflora* ‘Euiseong’ *P. lactiflora* ‘Sagok’	Si did not elevate stored carbohydrate levels over preservative-only treatments. The combination of Si and preservatives produced larger flowers. For ‘Taebaek’ peonies, SOD activity increased by 34.6% and 33.3% with Si added to water or preservative solution, respectively, and by 67.3% and 65.7% when Si was added to both, compared to their non-Si counterparts. For ‘Taebaek’: Delayed water loss started on day 5, significant decrease in fresh weight loss on day 8, SOD activity increased by 67.3% versus water and preservative treatment. For ‘Sagok’: Delayed water loss started on day 4, significant decrease in fresh weight loss on day 8, SOD activity decreased by 30.7% compared to water. For ‘Euiseong’: Delayed water loss started on day 6, significant decrease in fresh weight loss on day 6, ROS reduced by 49.7% versus water, 53.6% versus water + preservative.	[34] [35]

(Continued)

**Table 1:** Continued

Treatment	Concentration	Control	Cultivar	Effect	References
Calcium spray	4% CaCl_2_ solution, applied 21 days preharvest	Tap water	*P. lactiflora* ‘Yang Fei Chu Yu’	Extended vase life by 2.3 days.	[23]
NS + sucrose 8-HQ + S	1 mg·L^−1^ NS + 20 g·L^−1^ sucrose 200 mg·L^−1^ 8-HQC + 20 g·L^−1^ sucrose	Distilled water Distilled water	*P. lactiflora* ‘Charles Binder’ *P. lactiflora* ‘Wiesbaden’	Extended vase life (17%–35%), the lowest water uptake at 11.15 ± 3.92 g stem^−1^ day^−1^ for ‘Charles Binder’，the highest relative fresh weight at 133.37% ± 14.70%, indicating superior water retention, the lowest bacterial count at 2.20·10^−5^ CFU ml^−1^, suggesting effective reduction of bacterial blockages in ‘Charles Binder’. Extended vase life (only in ‘Wiesbaden’ by 32%), The relative fresh weight for ‘Charles Binder’ with the 8-HQC + S treatment was 154.55% ± 14.35%, which was the highest among the treatments.	[49] [49]
Sucrose Sucrose	20 g·L^−1^ 20 g·L^−1^	Deionized water Deionized water	*P. lactiflora* Yang Fei Chu Yu’ *P. lactiflora* ‘Yang Fei Chu Yu’	Extended vase life, increased flowering days, improved petal sugar content, and water absorption. Extended vase life by 1.8 days, from 6.2 days to 8 days, extended full bloom period by 1 day, from 3.3 days to 4.3 days.	[50] [51]
Trehalose	20 g·L^−1^	Deionized water	*P. lactiflora* ‘Hong Feng’	Extended vase life by 2.3 days, from 6.2 days to 8.5 days, extended full bloom period by 1.3 days, from 3.3 days to 4.6 days, flower diameter reached its maximum on the sixth day, indicating a delayed but prolonged full flowering period.	[51]
Glucose Glucose	20 g·L^−1^ 90 g·L^−1^	Deionized water Distilled water	*P. lactiflora* ‘Hong Feng’ *P.* × *suffruticosa* ‘Luoyang Hong’	Extended vase life by 1.4 days, from 6.2 days to 7.6 days, extended full bloom period by 0.7 days, from 3.3 days to 4 days. The 90 g·L^−1^ glucose treatment extended the vase life of *P. suffruticosa* ‘Luoyang Hong’ by 16.6 hours compared to the control, with a vase life of 92.8 hours versus 76.2 hours for the control. Additionally, it significantly delayed the flower opening process, showing a FOI of 1.8 at 8 hours compared to 2.9 for the control, and maintained a less senescent state (FOI of 5.7) at 96 hours compared to full senescence (FOI of 6.0) in the control.	[51] [52]

Ag-NPS, silver nanoparticles; ASA, acetylsalicylic acid; CA, calcium acetate; CaCl_2_, calcium chloride; CAT, catalase; CFU, colony-forming units; ClO_2_, chlorine dioxide; D-Arg, D-Arginine; FOI, flower opening index; H_2_O_2_, hydrogen peroxide; 8-HQC, 8-hydroxyquinoline citrate; MDA, malondialdehyde; MT, melatonin; NS, nanosilver; POD, peroxidase; ROS, reactive oxygen species; Si, silicon; SOD, superoxide dismutase.

‘N/A’ (not applicable) indicates that the data were not provided or is not relevant to the study context.

Control treatment: Typically refers to samples that receive no additional treatments or are treated only with distilled water, serving as a baseline for comparison.

**Table 2:** Ineffective and negative responses to preharvest and postharvest treatments in *Paeonia* cut flowers

**Treatment**	**Concentration**	**Control**	**Cultivar**	**Effect**	References
H_2_O_2_ H_2_O_2_	0.01 M 10 mmol·L^−1^	Distilled water Distilled water	*P.* × *suffruticosa* ‘Luoyang Hong’ *Paeonia lactiflora* ‘Da Fu Gui’	Vase life reduced by 1 day, SOD activity increased by 12.2%, CAT activity increased by 14.4%. Shortened vase life to 2.67 ± 0.81 days, decreased best viewing period to 2.33 ± 0.56 days, increased maximum flower diameter to 11.66 ± 0.41 cm. Water balance value reached zero 0.80 days earlier.	[31] [53]
NS + Sucrose	1 mg·L^−1^ NS + 2% or 4% sucrose	Distilled water	*P. lactiflora* ‘Sarah Bernhardt’	Not extend the vase life, but enhanced flower diameter, the increase in diameter relative to the respective water ranged from 15% to 33%, with the greatest increase for the NS + 4% sucrose solution (10.1 cm).	[55]
Sucrose Sucrose Sucrose	30 g·L^−1^ 60 g·L^−1^ 90 g·L^−1^	Distilled water Distilled water Distilled water	*P.* × *suffruticosa* ‘Luoyang Hong’ *P.* × *suffruticosa* ‘Luoyang Hong’ *P.* × *suffruticosa* ‘Luoyang Hong’	Produced a vase life of 77.1 hours, slightly longer than higher concentrations and marginally longer than the control at 76.2 hours, with a modest delay in flower opening that did not significantly improve overall vase longevity. Resulted in a vase life of 74.9 hours, shorter than the control’s 76.2 hours, and similarly to 30 g·L^−1^, it delayed flower opening without significantly benefiting overall vase life. Produced the shortest vase life of 74.8 hours, even though it delayed flower opening slightly more than the other concentrations, but this did not enhance vase life compared to the control’s 76.2 hours.	[52] [52] [52]
Glucose Glucose	60 g·L^−1^ 120 g·L^−1^	Distilled water Distilled water	*P.* × *suffruticosa* ‘Luoyang Hong’ *P.* × *suffruticosa* ‘Luoyang Hong’	Extended the vase life by 16.6 hours compared to the control, with a total of 92.8 hours versus 76.2 hours for the control. Significantly delayed the flower opening process, showing a FOI of 1.8 at 8 hours compared to 2.9 for the control. Maintained a less senescent state with an FOI of 5.0 at 48 hours and 5.7 at 96 hours, compared to full senescence (FOI of 6.0) in the control. Extended the vase life to 85.2 hours, offering less extension compared to 60 g·L^−1^ glucose but still an improvement over the control. Also delayed the flower opening process, with an FOI of 1.8 at 8 hours, similar to 60 g·L^−1^ but did not sustain as long, reaching an FOI of 5.8 at 96 hours. Showed a quicker approach to full senescence compared to 60 g·L^−1^ glucose, indicating diminishing returns at higher glucose concentrations.	[52] [52]

CAT, catalase; FOI, flower opening index; H_2_O_2_, hydrogen peroxide; NS, nanosilver; SOD, superoxide dismutase.

Control treatment: Typically refers to samples that receive no additional treatments or are treated only with distilled water, serving as a baseline for comparison.

## Sugar metabolism and its impact on senscence in *Paeonia*

Sugar metabolism plays a critical role in regulating the senescence of cut flowers of tree and herbaceous peonies, specifically affecting flower opening, delaying senescence, and modulating the antioxidant defense system.

### Impact of sugars on flower opening and lifespan

van Doorn et al. [54] suggested that petal senescence is related to sugar deficiency, and that the application of sugar to cut flowers significantly delays petal senescence. Zhang et al. [52] found that 90 g·L^−1^ glucose notably extended the vase life of *P.* × *suffruticosa* ‘Luoyang Hong’ cut flowers by about 10 hours longer than sucrose treatments. Xue et al. [50] reported that treating *P. lactiflora* ‘Yang Fei Chu Yu’ herbaceous peonies with 20 g·L^−1^ sucrose delayed the decrease in flower fresh weight by 1.3 days, maintaining a higher water content that is crucial for the decorative phase. Rabiza et al. [49] found similar beneficial effects on *P. lactiflora* ‘Wiesbaden’, where preservative solutions extended the vase life by up to 2 days by maintaining higher soluble sugars levels and improving water balance, compared to the control (distilled water). Similarly, Sun et al. [51] demonstrated that a preservative solution containing 20 g·L^−1^ of glucose significantly improved vase life across several herbaceous peony cultivars, with notable benefits in delaying blooming and senescence.

Skutnik et al. [55] observed that while nanosilver (NS) and sucrose treatments enhanced the aesthetic qualities of *P. lactiflora* ‘Sarah Bernhardt’ cut flowers, they did not significantly increase flower longevity. This indicates that while certain treatments may improve visual quality, they might not necessarily contribute to extending vase life, highlighting the need for precise optimization of treatment strategies.

### Expression of sugar metabolism-related genes

Sugar metabolism significantly influences senescence in tree and herbaceous peony cut flowers by regulating ETH production and gene expression. Zhang et al. [52] demonstrated that high concentrations of glucose and sucrose (90 g·L^−1^) initially suppressed ETH production and delayed its peak release, thereby extending the lifespan of *P.* × *suffruticosa* ‘Luoyang Hong’ petals by influencing genes linked to ETH biosynthesis. Similarly, a 60 g·L^−1^ glucose treatment further reduced ETH production by downregulating key genes for ACC synthase (ACS) and ETH response factor (ERF), critical for ETH signaling [56]. Moreover, during sucrose treatment at 20 g·L^−1^, the upregulation of sucrose transporter (SUT) and invertase (INV) genes enhanced the hydrolysis and transport of floral sugars, significantly prolonging the vase life of herbaceous peony *P. lactiflora* ‘Yang Fei Chu Yu’ [50]. These findings highlight the critical role of sugar metabolism in moderating flower senescence through hormonal regulation and gene expression.

### Sugar metabolism and postharvest vase flower quality

In terms of postharvest management, studies indicate that manipulating storage conditions, specifically dry storage and wet storage, can significantly influence vase quality by regulating starch and sugar metabolism. Dry storage (where cut flowers were kept out of water at 0–4°C) promoted better sugar utilization, which was crucial for maintaining vitality and delaying senescence in *P. lactiflora* ‘Yang Fei Chu Yu’ cut flowers [57]. On the other hand, wet storage (where stems were immersed in water during cold storage at 0–4°C) also played a role in determining the metabolic outcomes for peony cut flowers during and after storage [57].

For a detailed overview of the efficacy and drawbacks of various preharvest and postharvest treatments on sugar metabolism of peony cut flowers, refer to Tables 1 and 2.

## The impact of hormonal regulation on senescence in *Paeonia*

Plant hormones play a central regulatory role in the programmed senescence of plants. Through their complex signaling networks, these hormones precisely guide cell growth, differentiation, and ultimately death, directly determining the lifespan and quality of flowers [58]. Key hormones such as ETH, abscisic acid (ABA), gibberellins (GAs), cytokinins (CTKs), and auxins, like indole-3-acetic acid (IAA), coordinate the plant’s response to environmental signals through their internal regulatory networks [58,59].

### Ethylene

ETH plays a crucial role in plant senescence and is often referred to as the ‘senescence hormone’, while its role in regulating petal abscission, fruit ripening, and leaf yellowing has been widely studied [40].

In tree peony *P.* × *suffruticosa* ‘Luoyang Hong’, research by Zhou et al. [16] reported that 1-methylcyclopropene (1-MCP) significantly suppressed the expression of ETH receptor genes and biosynthetic genes like *PsACS1* and *PsACO1*, effectively reducing ETH production. This regulation of gene expression by 1-MCP inhibited ETH production and extended the vase life of cut flowers. Wang et al. [60] identified key EIN3-like genes (*PsEIL1*, *PsEIL2*, *and PsEIL3*) that are central to the ETH signaling pathway. These genes showed varied expression patterns related to different stages of *P.* × *suffruticosa* ‘Luoyang Hong’ development and senescence, with *PsEIL1* peaking during senescence, while *PsEIL2* and *PsEIL3* were most active during the flowering period. Wang et al. [61] observed that glucose not only delayed visible senescence in *P.* × *suffruticosa* ‘Luoyang Hong’ tree peony, but also suppressed the climacteric rise in ETH production by inhibiting ACC synthase activity. Their study noted a deviation from previous findings, focusing specifically on the inhibitory effects of glucose on ETH biosynthesis and its reduced sensitivity in tree peony. Furthermore, Wu et al. [62] explored the differential responses of *P.* × *suffruticosa* ‘Luoyang Hong’ and *P.* × *suffruticosa* ‘Xue Ying Tao Hua’ to ETH and its inhibitor 1-MCP. In *P.* × *suffruticosa* ‘Luoyang Hong’, ETH treatment significantly shortened vase life from 93.5 hours to 84.0 hours, while 1-MCP extended it to 101.1 hours. In contrast, *P.* × *suffruticosa* ‘Xue Ying Tao Hua’ exhibited a minimal response to both treatments, suggesting lower sensitivity to ETH.

**Figure 1:**
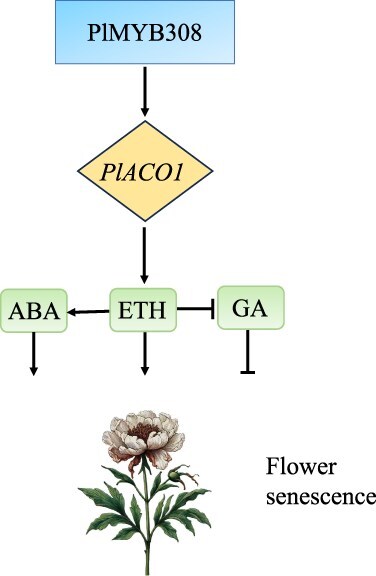
Regulatory Networks of Herbaceous Peony Flower Senescence. This figure illustrates the regulatory network involved in the senescence process of herbaceous peony flowers mediated by PlMYB308. PlMYB308 is a transcription factor that promotes the expression of *PlACO1* [63]. *PlACO1* acts downstream of PlMYB308 and influences the levels of ABA, ETH, and GA, ultimately regulating flower senescence

In the senescence of herbaceous peony cut flowers, PlMYB308 is a key transcription factor (TF) that is upregulated during the senescence of petals in *P.* × *suffruticosa* ‘Hangshao’ cut flowers. The expression of *PlMYB308* was induced by ETH and ABA and repressed by GAs. Silencing *PlMYB308* delayed flower senescence by reducing ETH and ABA levels and increasing GA levels, thus extending the flowering period [63]. This indicates that *PlMYB308* not only responds to ETH and ABA but also actively participates in their biosynthesis. Specifically, *PlMYB308* directly activates the promoter of *PlACO1*, an ETH biosynthetic gene, leading to increased ETH production, which subsequently promotes ABA accumulation and reduces GA levels. Therefore, *PlMYB308* serves as a crucial regulator in a positive feedback loop that enhances ETH and ABA production during flower senescence. This finding reveals the critical role of *PlMYB308* in ETH-regulated flower senescence (Fig. 1).

**Figure 2:**
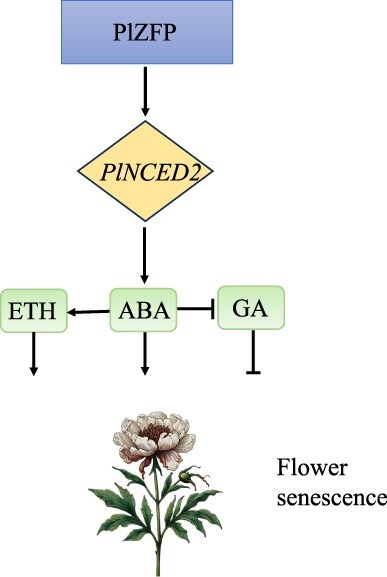
Regulatory Networks of Herbaceous Peony Flower Senescence. This figure depicts the regulatory network involved in the senescence process of herbaceous peony flowers mediated by *PlZFP*. *PlZFP* upregulates the expression of *PllNCED2*, a key enzyme in the ABA biosynthesis pathway, thereby influencing ETH and GA levels [15]. This network shows how *PlZFP* coordinates the interactions between ABA, ETH, and GA during flower senescence

### Abscisic acid

ABA is a crucial stress hormone in plants, playing a significant role in regulating plant growth, development, and environmental adaptability [40]. ABA’s role in plant senescence is particularly notable, mainly by regulating gene expression and signal transduction pathways that influence physiological changes [64].

For *P. lactiflora* ‘Hong Feng’ and other herbaceous peonies, ABA levels fluctuate throughout the flower’s life cycle, peaking during senescence, which suggests a strong link between ABA and the progression of senescence [22]. Another important discovery is the role of PlZFP, which is a zinc finger protein that regulates the senescence of herbaceous peony *P. lactiflora* ‘Hangshao’ cut flowers by mediating the interaction between ABA, GA, and ETH. *PlZFP* upregulated the expression of *PllNCED2*, a key enzyme in the ABA biosynthesis pathway, thereby affecting ETH production and GA levels [15]. This hormone interaction plays a crucial role in regulating flower senescence (Fig. 2).

### Gibberellins

GAs are vital for plant growth and development, influencing processes from cell expansion to senescence [58]. GA levels, particularly of GA_3_, significantly declined in herbaceous peony *P. lactiflora* ‘Qi Hua Lu Shuang’ as flowers aged, paralleling reductions in zeatin-riboside (ZR), another growth hormone [48]. Application of GA_3_ at 50 μM delayed petal senescence effectively, suggesting a crucial role in moderating the hormonal interplay that governs flower longevity [15].

GAs play a comprehensive role in maintaining the structural and aesthetic integrity of flowers postharvest by regulating genes and enzymes involved in cell division, elongation, and hormonal balance. This includes upregulating genes like GA20-oxidase and GA3-oxidase for GA production and downregulating GA2-oxidase for GA deactivation, balancing the processes that delay senescence and promote flower longevity [65]. These studies provide important insight into understanding how GAs might regulate the senescence of tree and herbaceous peony cut flowers, suggesting that GAs may delay senescence and extend flowering periods by modulating the balance of multiple hormone signals, although additional research is required to fortify this hypothesis.

### Cytokinins

CTKs play an essential role in plant cell division, differentiation, and senescence and by regulating the cell cycle, promoting protein synthesis, and delaying the senescence of leaves and flowers, CTKs significantly impact plant growth and development [21, 22].

In *P. lactiflora* ‘Hong Feng’ cut flowers, concentrations of CTKs like ZR and isopentenyl adenine (iPA) peak during the early color-showing stage, playing a crucial role in flower development and senescence. These hormones support cell elongation, nutrient transport, and balance ETH production, thus extending flower longevity [22]. Research in *P.* × *suffruticosa* ‘Hu Hong’ and *P.* × *suffruticosa* ‘Luoyang Hong’ indicates that ZR peaks at full bloom, correlating with delayed senescence and enhanced accumulation of storage substances, highlighting its significant role in floral longevity [42].

### Auxins

Auxins, particularly IAA, are crucial regulators of plant growth and development [66]. IAA not only promotes cell wall loosening and cell elongation but also impacts plant morphogenesis by finely regulating cell expansion, differentiation, and senescence [40].

The IAA efflux carrier gene, *PsPIN4*, identified in *P.* × *suffruticosa* ‘Xue Ying Tao Hua’ cut flowers, is crucial for petal abscission, where its expression increased with exogenous IAA, delaying abscission, and decreased with the auxin transport inhibitor 2,3,5-triiodobenzoic acid (TIBA), speeding up this process [67].

Zhao et al. [68] identified a genetic factor, tryptophan decarboxylase, in *P. lactiflora* ‘Da Fu Gui’, which plays a role in maintaining auxin levels under stress conditions, thereby enhancing drought and salt stress tolerance. This hormonal regulation is particularly important for supporting sustained physiological processes, such as cell expansion and growth, during environmental stress. This finding suggests that maintaining appropriate auxin levels is crucial for optimizing resilience and mitigating the effects of stress-induced senescence in peonies. Further research on a hybrid between the *P. lactiflora* ‘Fen Yu Nu’ and the *P.* ostii ‘Feng Dan Bai’ showed that IAA treatments enhanced the expression of the *PlARF2* gene, involved in auxin signaling, while ABA had a variable impact, highlighting the complex interactions between IAA and other hormones [69].

In summary, plant hormones play complex and crucial regulatory roles in the senescence of tree and herbaceous peonies (Tables 3 and 4).

**Table 3:** Quantified positive effects of hormonal treatments on peony flower senescence

**Hormone name**	**Treatment concentration**	**Cultivar**	**Effect**	References
ETH	ETH: 10 μmol·L^−1^	*P.* × *suffruticosa* ‘Luoyang Hong’	*PsEIL2* and *PsEIL3* mRNA increased 1.5-fold at 0, 12, and 24 hours.	[60]
ETH	ETH: 10 μmol·L^−1^	*P.* × *suffruticosa* ‘Luoyang Hong’	ETH treatment distinctly suppressed *Ps-ETR1-1* expression, maintaining about 25%–50% of mRNA levels of untreated control throughout the flower development stages; *Ps-EIN3-1* expression initially enhanced but then decreased to very low levels from preopening to half-opening, with a subsequent increase at full opening.	[16]
ETH	ETH: 10 μmol·L^−1^	*P.* × *suffruticosa* ‘Luoyang Hong’	Substantial stimulation of *PsACS1* mRNA expression was observed. Specifically, ETH treatment led to an immediate increase in *PsACS1* expression upon treatment, followed by gradual changes during different flower development stages, reaching a significantly higher expression level than untreated flowers at full opening.	[70]
ETH	Glucose: 90 g·L^−1^ ETH: 10 μmol·L^−1^	*P.* × *suffruticosa* ‘Luoyang Hong’	CK: Vase life began at 119 hours; GE: Extended vase life to 123.5 hours. FOI: At 48 hours, CK reached a FOI of 3.8. GE treatment led to an FOI of 4.0, indicating a slower senescence rate; EG treatment had an FOI similar to control at 4.0. ETH production: ETH production was initially enhanced in EG treatment compared to GE, but eventually, both treatments suppressed ETH production compared to the control.	[52]
ETH	Glucose: 60 g·L^−1^	*P.* × *suffruticosa* ‘Luoyang Hong’	ETH synthesis and signaling: Glucose treatment significantly down-regulated genes involved in ETH biosynthesis and signaling pathways. Specifically, one unigene encoding ACS, a key enzyme in ETH synthesis, and four unigenes encoding ERF, crucial in ETH signal transduction, were greatly repressed.	[56]
ETH	Untreated	*P.* × *suffruticosa* ‘Tao Hua Fei Xue’	ETH production: ETH production in the petals exhibited a climacteric peak during the opening stages, indicating a surge in ETH as the flower transitioned from preopening to full bloom. ACS activity: ACS activity closely followed the ETH production pattern, increasing as the flower approached full bloom, suggesting its role as a rate-limiting enzyme in ETH biosynthesis during senescence.	[47]
ETH	ETH: 10 ml·L^−1^ 1-MCP:1 ml·L^−1^	*P. suffruticosa* ‘Luoyang Hong’ *P. suffruticosa* ‘Luoyang Hong’	ETH treatment: accelerated ‘Luoyang Hong’ flower opening and senescence. 1-MCP treatment: postponed ‘Luoyang Hong’ flower senescence and extended vase life from 93.5 hours to 101.1 hours.	[62] [62]
ETH	ETH: 200 μM	*Paeonia lactiflora* ‘Hangshao’	ETH significantly accelerated senescence in herbaceous peony petals. Increased expression of *PlMYB308* associated with ETH treatment suggests a regulatory role in ETH-mediated senescence. ETH treatment also influenced other hormone levels and related gene expressions, integrating multiple hormonal pathways that affect senescence.	[63]
ABA	Light/moderate/severe shading: 80%/40%/20% sunlight	*P. ostii* ‘Feng Dan’	Light shading: decreased ABA concentration by 8.8%. Moderate shading: decreased ABA concentration by 14.4%. Severe shading: decreased ABA concentration by 22.7%.	[43]

(Continued)

**Table 3:** Continued

**Hormone name**	**Treatment concentration**	**Cultivar**	**Effect**	References
ABA	Severe shading: 30%–35% sunlight	*P. lactiflora* ‘Zi Feng Yu’ *P. lactiflora* ‘Hong Feng Yu’	Slight shading reduced the ABA content in leaves, which was associated with delaying senescence.	[46]
ABA	50 μmol·L^−1^	*P. lactiflora* ‘Fen Yu Nu’	Roots: At 50 μmol·L^−1^ ABA, *PlARF2* expression was initially higher than the CK (water treatment), then suppressed below CK levels as treatment duration increased. Leaves: *PlARF2* gene expression was continuously higher than the CK during treatment with 50 μmol·L^−1^ ABA, peaking at 2.78 times higher than CK at 4 hours.	[69]
GA	GA_3_: 50 μmol·L^−1^	*P. lactiflora* ‘Hangshao’	Delayed flower senescence, flowers remained in full bloom on the eighth day after treatment.	[15]
CTK	Untreated	*P. lactiflora* ‘Hong Feng’	The trends in iPA and ZR cytokinins differed. Both iPA and ZR were highest during the bud color showing stage. iPA levels decreased as the flower opened, peaked again during the initial opening stage, and then declined as aging accelerated. ZR levels dropped to their lowest at the initial opening stage and then slightly increased.	[22]
IAA	Untreated	*P. lactiflora* ‘Hong Feng’	IAA content in peony petals was highest during the bud color showing stage, reaching 365.1 ng·g^−1^ FW, then decreased to 126.5 ng·g^−1^ FW as the flower opened. During full bloom, IAA levels rose again, forming a peak. High initial levels of IAA promoted rapid petal elongation, with a surge at full bloom correlating with the onset of petal senescence.	[22]
IAA	IAA: 100 μmol·L^−1^	*P. lactiflora* ‘Fen Yu Nu’	With both concentrations of IAA, *PlARF2* expression in both roots and leaves was higher than water treatments (CK), with 100 μmol·L^−1^ showing slightly higher expression than at 50 μmol·L^−1^, suggesting positive regulation by IAA.	[69]

ABA, abscisic acid; ACS, ACC Synthase; CK, control; CTK, cytokinins; EG, ethylene then glucose; ERF, ethylene response factor; ETH, ethylene; FOI, flower opening index; FW, fresh weight; GA, gibberellin; GE, glucose then ethylene; IAA, auxins; iPA, isopentenyladenine; 1-MCP, 1-methylcyclopropene; ZR, zeatin riboside.

Control treatment: typically refers to samples that receive no additional treatments or are treated only with distilled water, serving as a baseline for comparison.

## The impact of environmental factors on senescence in *Paeonia*

### Light and temperature

Environmental factors, especially light and temperature, significantly influence the senescence of tree and herbaceous peonies.

#### The impact of light and temperature fluctuations

Shading treatments significantly delay the senescence of tree and herbaceous peonies by reducing light-induced oxidative stress and improving stress resistance and productivity [43,46]. In tree peony *P. ostii* ‘Feng Dan’, reducing solar exposure to about 80% notably improved photosynthetic efficiency and reduced oxidative stress, delaying leaf senescence and enhancing seed quality [43]. Similarly, for herbaceous peonies *P. lactiflora* ‘Zi Feng Yu’ and *P. lactiflora* ‘Hong Feng Yu’, slight shading (reducing solar exposure to about 65-70%) increased chlorophyll content and photosynthetic rates, contributing to prolonged leaf lifespan and overall plant health [46].

High temperatures (about 28°C) accelerated senescence in *P.* × *suffruticosa* ‘Lu He Hong’ tree peony by significantly altering protein and hormone profiles, with an increase in ABA and a decrease in IAA. This thermal stress led to quicker petal abscission, indicating temperature’s critical impact on hormonal regulation and petal longevity [71]. In a study by Zu et al. [17], *P. lactiflora* ‘Chi Fen’ and *P. lactiflora* ‘Meigui Zi’ exhibited varying responses to high temperatures, demonstrating the impact of genetic differences on stress tolerance. *P. lactiflora* ‘Chi Fen’, tolerant to heat, maintained higher photosynthetic efficiency and chlorophyll content at temperatures ranging from 32°C to 34°C, while *P. lactiflora* ‘Meigui Zi’ was more susceptible to heat-induced premature aging. This was evident in their differential gene expression related to photosynthesis and stress responses, including photosystems I and II, electron transport, and light-harvesting complexes. These findings highlight the genetic basis behind the varying abilities of peony cultivars to withstand high temperatures, significantly affecting their senescence patterns [17].

**Table 4:** Ineffective and negative responses to hormonal treatments in peony flower senescence

**Hormone name**	**Treatment concentration**	**Cultivar**	**Effect**	References
ETH	1-MCP: 1 μL·L^−1^	*P.* × *suffruticosa* ‘Luoyang Hong’	1-MCP: no significant change in *PsEIL1* mRNA levels. *PsEIL3* decreased 4-fold by 12 hours and 2.5-fold by 24 hours.	[60]
ETH	1-MCP: 1 μL·L^−1^	*P.* × *suffruticosa* ‘Luoyang Hong’	1-MCP: inhibited *Ps-ETR1-1* mRNA expression, which decreased gradually during flower opening from stages 1 to 4, and increased slightly when the flower fully opened at stage 5, but caused a slight increase in *Ps-EIN3-1* transcript levels throughout the flower development, similar to but less than the changes observed with ETH treatment.	[16]
ETH	1-MCP: 1 μL·L^−1^	*P.* × *suffruticosa* ‘Luoyang Hong’	1-MCP: inhibited *PsACS1* mRNA expression. 1-MCP treatment led to a decrease in *PsACS1* expression throughout the flower’s vase life, with only faint mRNA signals detected in petals of fully opened flowers. *PsACO1* expression: there was no significant alteration in the accumulation of *PsACO1* transcript in the petals across different treatment conditions, indicating a more constitutive expression pattern unaffected by ETH and 1-MCP.	[70]
ETH	Glucose: 90 g·L^−1^	*P.* × *suffruticosa* ‘Luoyang Hong’	CK: vase life began at 119 hours. EG: Vase life was 113.6 hours.	[52]
ETH	Glucose: 90 g·L^−1^	*Paeonia lactiflora* ‘Tao Hua Fei Xue’	ETH production: while the increase in ETH supported flowering processes, it also signified the onset of senescence, marking a transition towards the end of the flower’s lifecycle. Respiration rate: a significant peak in respiration rates in petals was noted, which corresponded with ETH production peaks.	[47]
ETH ETH	ETH: 10 ml·L^−1^ 1-MCP: 1 ml·L^−1^	*P.* × *suffruticosa* ‘Luoyang Hong’ *P.* × *suffruticosa* ‘Xue Ying Tao Hua’	In ‘Luoyang Hong’, ETH treatment reduced vase life from 93.5 hours to 84.0 hours, indicating a negative effect on longevity despite promoting faster opening. ‘Xue Ying Tao Hua’ showed no sensitivity to ETH; vase life and flower development stages were not significantly affected by either ETH or 1-MCP.	[62] [62]
ABA	ABA: 100 μmol·L^−1^	*P. lactiflora* ‘Hangshao’	Increased expression of *PlMYB308*, a gene associated with the senescence of herbaceous peony flowers. This treatment accelerated flower senescence.	[63]
ABA	ABA: 100 μmol·L^−1^	*P. lactiflora* ‘Hangshao’	Increased *PlZFP* transcript abundance, promoting senescence in peony flowers. Also used in combination with Ethephon for synergistic effects on senescence.	[15]
ABA	ABA: 100 μmol·L^−1^	*P. lactiflora* ‘Fen Yu Nu’	Roots: at 100 μmol·L^−1^, *PlARF2* expression in both roots and leaves was lower than at 50 μmol·L^−1^.	[69]

ABA, abscisic acid; CK, control; EG, ethylene then glucose; ETH, ethylene; FOI, flower opening index; GE, glucose then ethylene; 1-MCP, 1-methylcyclopropene.

Control treatment: typically refers to samples that receive no additional treatments or are treated only with distilled water, serving as a baseline for comparison.

Conversely, low temperatures effectively delayed senescence. Sun et al. [51] demonstrated that a controlled atmosphere with 5% O_2_ and 20% CO_2_, combined with a low temperature of 2°C, significantly extended the vase life of herbaceous peony cultivars, such as *P. lactiflora* ‘Dan Feng’, *P. lactiflora* ‘Lu Xihong’, *P. lactiflora* ‘Qihua Lushuang’, *P. lactiflora* ‘Lian Tai’, and *P. lactiflora* ‘Hong Feng’. This environment slowed respiration and tissue degradation, outperforming cold storage alone. The study emphasized that synergistic conditions of low temperature and specific gas concentrations effectively enhance cut flower preservation by maintaining protective enzyme activity and reducing oxidative damage, thereby improving postharvest freshness.

#### The impact of dry and wet storage

Tang et al. [72] studied the effects of dry and wet storage on the postharvest quality of herbaceous peony *P. lactiflora* ‘Yang Fei Chu Yu’ at 4°C, using treatments including direct dry storage, dry storage with a preliminary 6-hour ‘Chrysal’ (a commercial floral preservative) treatment, continuous wet storage in water, and wet storage preceded by ‘Chrysal’. After storage, cut flower quality was assessed over 3 and 23 days at room conditions. Results indicated that dry storage maintained higher soluble sugar levels in petals compared to wet storage. Although ‘Chrysal’ was used, it did not significantly affect the soluble sugar or protein content in petals, suggesting limited enhancement in long-term preservation. This highlights the importance of further research into optimizing storage conditions and preservative formulations to improve the longevity and marketability of cut flowers like *P. lactiflora* ‘Yang Fei Chu Yu’.

### The impact of cultivation practices on senescence in *Paeonia*

Cultivation practices, such as soil management and fertilization, significantly impact the development and senescence of peonies. Sun et al. [73] demonstrated that the application of carbon-based nutrient fertilizers, in combination with chemical fertilizers, improved soil fertility, enhanced seed yield, and promoted the establishment of beneficial microbial communities in *P. ostii* ‘Feng Dan’. These improvements were associated with increased organic matter and nutrient availability, which are critical factors in delaying senescence and improving overall plant health.

Similarly, Joshi et al. [74] evaluated the impact of different organic fertilizers, specifically farmyard manure and vermicompost, on *Podophyllum emodi*. The study found that vermicompost, especially when applied to raised beds, significantly improved soil moisture, organic carbon content, and overall plant growth, including increased biomass and production of secondary metabolites. These findings indicate that optimized organic fertilization and bed preparation are effective strategies to enhance peony growth, potentially delaying senescence by improving nutrient uptake and stress resilience.

Existing research provides insights into how environmental factors affect the senescence of tree and herbaceous peonies (Tables 5 and 6).

**Table 5:** Beneficial effects of environmental treatments on peony cultivar performance

**Environmental factor**	**Treatment**	**Cultivar**	**Effect**	References
Light intensity	Light shading	*P. ostii* ‘Feng Dan’	Increased seed yield by 9.7%, increased crude fat by 5.6%, and increased unsaturated fatty acids by 9.6% compared to nonshaded plants.	[43]
Light intensity Light intensity	Slight shading Slight shading	*Paeonia lactiflora* ‘Zi Feng Yu’ *P. lactiflora* ‘Hong Feng Yu’	Delayed leaf senescence, increased net photosynthetic rate, increased chlorophyll and soluble sugar content, reduced proline, and abscisic acid content. Leaf senescence prolonged by 10 days. Leaf senescence delayed by 20 days, demonstrating slightly better resistance to strong light intensity compared to ‘Zi Feng Yu’.	[46] [46]
Temperature	Room temperature	*P.* × *suffruticosa* ‘Lu He Hong’	Maintained longer flowering period, less significant changes in ABA and IAA levels compared to high temperature.	[71]
Harvest stage Harvest stage Harvest stage Harvest stage	SB stage UT stage TB stage CC stage	*P. lactiflora* ‘Dan Feng’ *P. lactiflora* ‘Lu Xihong’ *P. lactiflora* ‘Qihua Lu shuang’ *P. lactiflora* ‘Lian Tai’	Highest flowering rate (100%), longest vase life (7.6 days). Flowering rate of 100%, full blooming time, and vase life of 7.2 days. Lower flowering rate (80%), vase life of 7.8 days. Highest flowering rate (100%), longer blooming period (7.5 days).	[51] [51] [51] [51]

ABA, abscisic acid; CC stage, changing-color; IAA, auxins; Light shading, 80% sunlight; room temperature, 20°C; SB stage, soft-bud; slight shading, 30%–35% sunlight; TB stage, tight-bud; UT stage, unfold-top.

Control treatment: typically refers to samples that receive no additional treatments or are treated only with distilled water, serving as a baseline for comparison.

## Future research directions

### Mechanistic approaches for enhancing preservation techniques in *Paeonia*

**Figure 3:**
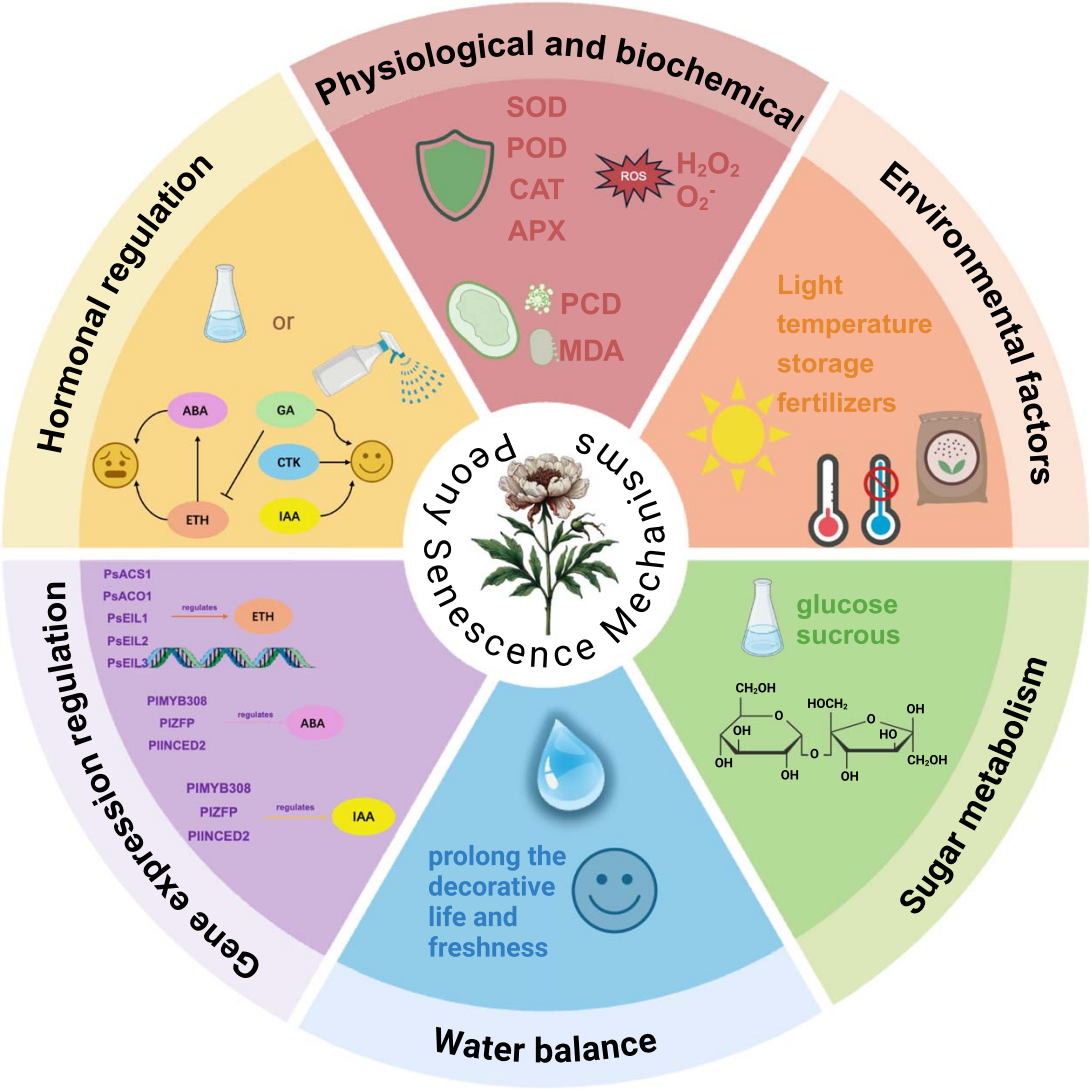
Key factors influencing the senescence of peony flowers. This figure illustrates the key factors influencing the senescence of peony flowers, organized into six main interconnected modules: 1. Physiological and biochemical changes: This module includes antioxidant enzymes such as SOD, POD, CAT, and APX, which help mitigate oxidative stress. ROS, H_2_O_2_, and O_2_^−^ are highlighted as stress indicators, while PCD and MDA represent markers of cellular damage during senescence. 2. Environmental factors: External elements like light, temperature, storage conditions, and fertilizers influence the regulation of hormonal levels and subsequently affect the senescence and longevity of peony flowers. 3. Sugar metabolism: Sugars, including glucose and sucrose, serve as essential energy sources, maintaining cellular activity and supporting the structural integrity of flowers during senescence. 4. Water balance: Adequate water availability is crucial in delaying senescence and extending the decorative lifespan of peony flowers, represented here by symbols indicating water maintenance and freshness. 5.Gene expression regulation: Key genes involved in hormonal signaling include *PsACS1*, *PsACO1*, *PsEIL1*, *PsEIL2*, *PsEIL3*, *PlMYB308*, *PlZFP*, *PllNCED2*, *PsPIN4*, and *PlARF2*. These genes regulate hormone biosynthesis and signaling, impacting senescence through interactions with ETH, ABA, and IAA pathways. 6. Hormonal regulation: The interplay between major hormones—ETH, ABA, GA, CTK, and IAA—is shown, illustrating their collective influence on flower senescence. ETH is prominently featured for its role in promoting senescence, while other hormones interact to delay or enhance this process. The central circle represents the overall ‘Peony Senescence Mechanisms’, with each module interacting to demonstrate the complexity of factors contributing to the aging of peony flowers. The figure was created using both BioRender and PowerPoint software

To further enhance floral longevity and understand plant aging processes, future research should focus on exploring the intricate mechanisms by which treatments such as sucrose, MT, and silicon influence flower senescence. Detailed mechanistic studies of antioxidant responses, water metabolism, and energy regulation will help refine these preservation techniques, optimize treatment concentrations, and establish new protocols for managing postharvest senescence. These efforts could ultimately improve the ornamental value and market competitiveness of tree and herbaceous peonies (Fig. 3).

**Table 6:** Ineffective and negative impacts of environmental treatments on peony cultivar outcomes

**Environmental factor**	**Treatment**	**Cultivar**	**Effect**	References
Light intensity Light intensity	Moderate shading Severe shading	*P. ostii* ‘Feng Dan’ *P. ostii* ‘Feng Dan’	Decreased seed yield by 9.8%, crude fat by 4.8%, and unsaturated fatty acids by 3.2% in 2015; similar trends observed in 2016. Decreased seed yield by 33.3%, crude fat by 7.7%, and unsaturated fatty acids by 6.3% in 2015; similar trends observed in 2016.	[43] [43]
Drought	Withheld watering to simulate natural drought conditions	*Paeonia lactiflora* ‘Da Fu Gui’	REC increased by 82.87% on day 21, and MDA levels increased by 160% on day 21, indicating heightened oxidative stress. Proline content increased 13-fold compared to control on day 21, a response to osmotic stress, significant accumulation of H_2_O_2_ and O_2_^−^ noted on day 21.	[68]

H_2_O_2_, hydrogen peroxide; MDA, malondialdehyde; moderate shading, 40% sunlight; O_2_^−^, superoxide anion; REC, relative electrical conductivity; severe shading, 20% sunlight.

Control treatment: typically refers to samples that receive no additional treatments or are treated only with distilled water, serving as a baseline for comparison.

### Exploration of hormonal interactions in *Paeonia* senescence

Plant hormones such as ETH, GAs, CTKs, and IAA play critical roles in regulating flower senescence in tree and herbaceous peonies. Existing studies suggest that GA_3_ application has the potential to extend vase life and enhance antioxidant enzyme activities in peonies [65], similar to its effects in other ornamental plants like daffodils (*Narcissus pseudonarcissus* L. ‘Dutch Master’) and *Anthurium andraeanum* [75, 76]. ETH’s role in regulating senescence has been observed in cut roses (*Rosa hybrida* ‘Samantha’) through its influence on genes involved in petal expansion and hormone receptor degradation, which highlights a potential mechanism applicable to peonies [77–79]. CTKs and IAA are also crucial. IAA treatment in a hybrid of *P. lactiflora* ‘Fen Yu Nu’ and *P. ostii* ‘Feng Dan Bai’ enhanced auxin signaling, while CTKs in other species have been shown to delay senescence [69, 80, 81]. Future research should focus on exploring how these hormones interact under different environmental conditions to optimize postharvest treatments and extend flower longevity.

### Application of artificial light sources in *Paeonia* postharvest management

Managing light conditions, such as through shading, has been beneficial in delaying senescence in peonies, but the potential of artificial light sources, such as light-emitting diode (LED)-derived lighting, remains largely unexplored. LED lights offer adjustable light quality and intensity, which could be advantageous for delaying senescence under controlled conditions. Although there is currently a lack of direct research on the effects of LED lighting on peonies, studies in other plant species have shown promising results. For instance, in lettuce (*Lactuca sativa* L.), a combination of red and blue LEDs at a ratio of 7:3 reduced transcriptome differences between young and old leaves, indicating delayed aging [82]. Moreover, Shi et al. [83] demonstrated that using white LEDs combined with bacterial volatile organic compounds successfully maintained the green state of broccoli (*Brassica oleracea var. italica*) and delayed its aging process, suggesting that artificial light treatments can significantly impact postharvest quality. Extending similar research to peonies may provide insight into how artificial lighting can be used to enhance postharvest quality and delay senescence, particularly in environments where natural light is not ideal.

### Impact of environmental stress factors on *Paeonia* senescence

Besides light and temperature, environmental stress factors such as drought, salt, and biotic stress are known to significantly influence plant senescence by increasing oxidative stress and disrupting physiological processes. While these effects have been documented in various species, studies specific to peonies are still limited. For instance, drought stress in herbaceous peony *P. lactiflora* ‘Da Fu Gui’ has been linked to increased MT production, which enhances stress tolerance [68]. Similarly, jasmonic acid (JA), which is known for its complex role in stress response, has shown potential in delaying or accelerating senescence depending on environmental conditions and plant species [84,85]. Research into the mechanisms by which these stressors affect peony senescence could help develop more effective stress management strategies, ultimately extending floral longevity and improving resilience.

### Expanding the molecular regulatory network in *Paeonia* senescence

Flower senescence is the final process in flower development, characterized by fading, wilting, and abscission [58]. These changes are regulated by genes and developmental controls [19]. Petal senescence, a key factor affecting the quality of ornamental plants, is controlled by a combination of gene expression and plant hormone signaling [58].

#### Key TFs

Understanding the roles of TFs in senescence is pivotal. In peonies, TFs like PlMYB308 have been found to regulate senescence-related pathways [63]. Insights from other species suggest that TFs like *ERFs* and *WRKY* are involved in regulating senescence-related gene expression, suggesting that similar regulatory mechanisms might also be crucial for peonies [86–88]. Future research should specifically investigate how these TFs interact within the unique physiological context of tree and herbaceous peonies, and under which environmental conditions these TFs could be manipulated to develop effective flower preservation strategies.

#### Epigenetics

Epigenetic regulation, such as histone modifications, also plays a significant role in plant senescence. While specific studies on epigenetic regulation in peony senescence are limited, similar findings in cut carnation (*Dianthus caryophyllus* ‘Master’) indicate that *H3K4* methylation affects senescence-related genes [10]. Investigating the role of histone modifications and other epigenetic changes specifically in peonies could provide insights into senescence regulation and help develop targeted approaches to extend flower longevity.

#### Noncoding RNAs

Noncoding RNAs, such as miRNAs and lncRNAs, play crucial roles in regulating plant senescence. In *P. ostii* ‘Feng Dan’, integrated analyses involving the miRNAome, transcriptome, and degradome identified significant miRNA-mRNA interactions that control floral development and senescence, targeting key TFs like MYB, bHLH, and NAC [14]. These findings provide an important basis for expanding research on the regulatory roles of noncoding RNAs in both tree and herbaceous peonies, with a focus on how manipulating these interactions could enhance flower quality and vase life.

#### Transmembrane receptors and second messenger systems

Transmembrane receptors and secondary messenger systems are crucial for transmitting senescence signals in plants. In peonies, potential signaling pathways involving *ETH* and downstream *MAPK* cascades need to be investigated to understand their roles in petal senescence, particularly for extending postharvest quality [58]. Exploring whether these signaling components exist and function similarly in peonies will provide valuable insights for optimizing postharvest treatments and improving flower longevity.

### Technological innovations in *Paeonia* senescence research

#### Synthetic biology

The rapid development of gene editing technologies, particularly CRISPR/Cas9, has provided an unprecedented opportunity to precisely modify genes that regulate plant traits, including senescence. In tree and herbaceous peonies, this technology holds potential for improving flower lifespan and stress tolerance by targeting specific genes involved in senescence.

Liu et al. [89] identified senescence-associated genes in *P.* × *suffruticosa* ‘Lu He Hong’, such as isocitrate dehydrogenase and ATP synthase subunit beta, through suppression subtractive hybridization (SSH). These genes are potential targets for CRISPR/Cas9 editing to delay flower senescence. For example, precise editing of isocitrate dehydrogenase could alter cellular energy metabolism, reducing oxidative stress and extending flower life.

In *Petunia hybrida* ‘Mirage Rose’, CRISPR/Cas9 editing of the *PhACO1* gene, involved in ETH biosynthesis, reduced ETH production and extended flower lifespan [90]. A similar approach could be applied to peonies to target ETH biosynthesis pathways, particularly considering existing findings on *PlMYB308* in peonies. The expression of *PlMYB308* was induced by ETH and ABA and repressed by GAs, and silencing *PlMYB308* delayed flower senescence by reducing ETH and ABA levels and increasing GA levels, thereby extending the flowering period [63]. Specifically, *PlMYB308* directly activates the promoter of *PlACO1*, an ETH biosynthetic gene, leading to increased ETH production, which promotes ABA accumulation and reduces GA levels. Targeting *PlMYB308* or *PlACO1* using CRISPR/Cas9 could potentially break this positive feedback loop, reduce ETH and ABA levels, and delay senescence in peonies.

Furthermore, editing *SWEET* genes, which are involved in sugar transport, may enhance stress tolerance in peonies. By enhancing *SWEET* gene expression, peonies could better manage water and energy, reducing the impact of drought and heat stress, ultimately extending their ornamental value [91].

These examples highlight that CRISPR/Cas9 can be used to modify specific genes regulating senescence, stress tolerance, and hormone pathways in peonies. Future studies should include gene knock-in and knock-out experiments to understand the roles of these genes precisely, enabling effective genetic interventions for improved flower quality and longevity.

#### Integrated multi-omics studies

Integrated multi-omics approaches, combining transcriptomics, proteomics, and metabolomics, provide comprehensive insights into the molecular mechanisms underlying plant senescence. These technologies are particularly relevant for tree and herbaceous peonies, where a deep understanding of senescence at the molecular level is still emerging.

Transcriptomics reveals gene expression changes that are crucial for identifying key regulators of senescence. In *P.* × *suffruticosa* ‘Lu He Hong’, transcriptomics and SSH identified several differentially expressed genes involved in hormone regulation and signal transduction during the transition from blooming to senescence [89]. These findings provide a foundation for understanding key genetic players in peony senescence, and future research could focus on applying this understanding to genetic interventions aimed at extending flower lifespan.

Proteomics analyzes protein modifications and interactions, providing insights into protein-level changes during senescence. Comparative proteomics studies in lily (*Lilium* spp.) identified proteins like ATP synthase CF1 alpha subunit (AtpA) as key factors in regulating vase life under different treatments [92]. Similar proteomic analyses in peonies could help understand protein dynamics and identify candidates that affect senescence.

Metabolomics sheds light on the role of metabolites in regulating senescence. For example, studies in petunia (*P. hybrida*) revealed that altering the acetyl-CoA pathway significantly influenced flower senescence by modifying metabolite levels [93]. In peonies, similar metabolomic profiling could help elucidate how changes in sugars, amino acids, and other small molecules impact senescence, offering opportunities for targeted interventions.

Applying integrated multi-omics studies that combine transcriptomics, proteomics, and metabolomics to tree and herbaceous peonies might be able to provide a holistic understanding of the molecular basis of flower senescence. This could lead to the identification of new regulatory targets for breeding and postharvest treatments, ultimately extending flower longevity and enhancing ornamental value. Future research should focus on utilizing these technologies to explore senescence mechanisms under various environmental conditions, providing a foundation for sustainable horticultural practices.

These technologies are pivotal for understanding the complex biological processes in tree and herbaceous peonies, especially during their senescence phases.

#### Space biology

Research into the effects of microgravity on plant biology has revealed significant changes in cellular processes and gene regulation that affect plant growth and adaptation. Studies indicate that microgravity alters cell proliferation, differentiation, and gene regulatory mechanisms, such as regulatory protein activity, microRNA function, and DNA chemical modifications [94]. These insights are crucial for understanding plants’ gravity sensing and response mechanisms.

For tree and herbaceous peonies, conducting long-term growth experiments in microgravity could offer new perspectives on plant senescence. Specifically, assessing physiological and biochemical markers such as antioxidant enzyme activity, hormone levels, and gene expression in microgravity compared to ground controls could help identify unique regulatory factors affecting senescence that differ from terrestrial responses. Targeting key factors influenced by space conditions could lead to the development of new methods for enhancing plant resilience and preservation techniques on Earth, especially for ornamental plants like peonies.

### Expanding research areas

#### Autophagy

Autophagy is a crucial intracellular process that regulates plant senescence by recycling cellular components. Although the specific role of autophagy in tree and herbaceous peonies remains underexplored, high-throughput sequencing could help identify and characterize key autophagy-related genes like *ATG8*, which are known to play essential roles in regulating senescence and maintaining cellular homeostasis in other species [95]. In *Arabidopsis* and tomato (*Solanum lycopersicum* ‘Ailsa Craig’), autophagy genes such as *ATG6* and *ATG5* have been shown to influence plant responses to stress and pathogen resistance [96, 97]. This suggests that similar genes in peonies may help mitigate stress-induced senescence. Leveraging CRISPR/Cas9 technology to edit autophagy-related genes could provide deeper insights into their regulatory functions, potentially extending flower longevity and improving postharvest quality.

#### Global climate change

In the face of global climate change, it is necessary to simulate different environmental stresses, such as increased temperatures and altered precipitation, to understand their physiological and molecular impacts on in planta tree and herbaceous peonies. Specifically, targeted analyses of growth indicators, cellular senescence markers, and the expression of climate-related adaptive genes could provide valuable information for developing new climate-resilient peony varieties. For example, increased temperatures may accelerate the senescence process, while altered precipitation could impact water uptake and hormonal balance. By focusing on these key physiological and molecular changes, researchers can identify adaptive traits that help peonies thrive under unconventional climate conditions, contributing to the development of effective preservation strategies and the enhancement of their ornamental value [98].

## Conclusion

This review summarizes the research progress on the senescence mechanisms of tree and herbaceous peonies, focusing on gene expression, hormone interactions, and environmental factors. Key genes like *PlMYB308* and *PlZFP* play essential roles in regulating hormone signaling pathways, which influence the onset of senescence. Hormones, such as ETH, ABA, GAs, CTKs, and IAA, are central to coordinating these processes. Moreover, maintaining appropriate antioxidant levels, water balance, and energy metabolism are critical factors in delaying senescence.

Despite the progress made, significant challenges remain. There is still much to understand regarding the differential responses among peony varieties to environmental stresses, and how these stresses influence senescence. The complexity of hormone regulatory networks under varying environmental conditions requires further exploration to improve stress resilience and prolong the flowering period in different peony varieties.

Future research should focus on utilizing advanced technologies, including CRISPR/Cas9 for gene editing, and multi-omics approaches—such as transcriptomics, proteomics, and metabolomics—to further elucidate senescence mechanisms. Special attention should be given to understanding autophagy and programmed cell death, as well as their roles in senescence regulation. Additionally, new strategies like LED lighting for environmental control, and exploring hormone interactions at the molecular level, can significantly contribute to enhancing the ornamental and commercial value of peonies.

Integrating these diverse research approaches will support the development of more resilient peony varieties and optimize postharvest treatments, contributing significantly to both the floriculture industry and our broader understanding of plant senescence mechanisms.

## References

[1] Yu XN . Ornamental Peony. Beijing: China Forestry Publishing House; 2019: (in Chinese)

[2] Li JY . Chinese Tree Peonies and Herbaceous Peonies. China Forestry Publishing House; 1996: (in Chinese)

[3] Tian Y, Wang XNL. Let the "national flower peony" bloom more vigorously and brilliantly. Henan Daily (Nat Sci). 2023;008. (in Chinese)

[4] Wang YX, Yu XN. The glory of herbaceous peony—the revival and opportunities of traditional famous flowers. China Flowers & Horticulture (Nat Sci). 2024;2:36–9 (in Chinese)

[5] Kamenetsky-Goldstein R, Yu X. Cut peony industry: the first 30 years of research and new horizons. Hortic Res. 2022;9:uhac079 35669702 10.1093/hr/uhac079 PMC9157654

[6] Teixeira da Silva JA . The cut flower: postharvest considerations. Online J Biol Sci. 2003;3:406–42

[7] Sade N, Del Mar R-WM, Umnajkitikorn K. et al. Stress-induced senescence and plant tolerance to abiotic stress. J Exp Bot. 2018;69:845–53 28992323 10.1093/jxb/erx235

[8] Ay N, Janack B, Humbeck K. Epigenetic control of plant senescence and linked processes. J Exp Bot. 2014;65:3875–87 24683182 10.1093/jxb/eru132

[9] Feng S, Jiang X, Huang Z. et al. DNA methylation remodeled amino acids biosynthesis regulates flower senescence in carnation (*Dianthus caryophyllus*). New Phytol. 2024;241:1605–20 38179647 10.1111/nph.19499 PMC11806656

[10] Feng S, Jiang X, Wang R. et al. Histone *H3K4* methyltransferase *DcATX1* promotes ethylene induced petal senescence in carnation. Plant Physiol. 2023;192:546–64 36623846 10.1093/plphys/kiad008 PMC10152666

[11] Chen WH, Lin PT, Hsu WH. et al. Regulatory network for FOREVER YOUNG FLOWER-like genes in regulating Arabidopsis FLOWER senescence and abscission. Commun Biol. 2022;5:662 35790878 10.1038/s42003-022-03629-w PMC9256709

[12] Cheng SLH, Xu H, Ng JHT. et al. Systemic movement of long non-coding RNA ELENA1 attenuates leaf senescence under nitrogen deficiency. Nat Plants. 2023;9:1598–606 37735255 10.1038/s41477-023-01521-x

[13] Holme IB, Ingvardsen CR, Dionisio G. et al. CRISPR/Cas9-mediated mutation of *Eil1* transcription factor genes affects exogenous ethylene tolerance and early flower senescence in *campanula portenschlagiana*. Plant Biotechnol J. 2024;22:484–96 37823527 10.1111/pbi.14200 PMC10826993

[14] Guo L, Li Y, Zhang C. et al. Integrated analysis of miRNAome transcriptome and degradome reveals miRNA-target modules governing floral florescence development and senescence across early- and late-flowering genotypes in tree peony. Front Plant Sci. 2022;13:1082415 36589111 10.3389/fpls.2022.1082415 PMC9795019

[15] Ji XT, Yuan YP, Bai ZZ. et al. PlZFP mediates the combinatorial interactions of abscisic acid with gibberellin and ethylene during flower senescence in cut herbaceous peony. Postharvest Biol Technol. 2023;195:112130.

[16] Zhou L, Dong L, Jia PY. et al. Expression of ethylene receptor and transcription factor genes, and ethylene response during flower opening in tree peony (*Paeonia suffruticosa*). Plant Growth Reg. 2010;62:171–9

[17] Zu MT, Qiu SY, Qian Y. et al. Transcriptome sequencing provides insights into high-temperature-induced leaf senescence in herbaceous peony. Agriculture. 2024;14:547

[18] Locato V, De Gara L. Programmed cell death in plants: an overview. Methods Mol Biol. 2018;1743:1–8 29332281 10.1007/978-1-4939-7668-3_1

[19] van Doorn WG, Woltering EJ. Physiology and molecular biology of petal senescence. J Exp Bot. 2008;59:453–80 18310084 10.1093/jxb/erm356

[20] Xu L, Sun M, Teixeira da Silva JA. et al. Assessment of programmed cell death in aging *Paeonia lactiflora* petals. Cytologia. 2023;88:151–9

[21] Tong N, Shu Q, Wang B. et al. Histology, physiology, and transcriptomic and metabolomic profiling reveal the developmental dynamics of annual shoots in tree peonies (*Paeonia suffruticosa* Andr.). Hortic Res. 2023;10:uhad152. 37701456 10.1093/hr/uhad152 PMC10493643

[22] Shi GA, Guo XF, Zhang GH. et al. Changes in physiological indicators during the opening and senescence of herbaceous peony flowers. Acta Bot Boreali-Occidentalia Sin (Nat Sci). 2008;3:506–11 (in Chinese)

[23] Hui GJ, Zheng GS, Zhang W. et al. Effects of pre-harvest calcium spray on the physiological and biochemical characteristics of herbaceous peony cut flowers. Acta Bot Boreali-Occidentalia Sin (Nat Sci). 2009;29:1246–51 (in Chinese)

[24] Wang X . Effects of Pre-Harvest Spraying of Calcium and Bud Removal Interactions on the Quality of Cut Peonies (MSc thesis). Beijing: Chinese Academy of Forestry Sciences; 2016: (in Chinese)

[25] Zhao D, Luan Y, Shi W. et al. Melatonin enhances stem strength by increasing lignin content and secondary cell wall thickness in herbaceous peony. J Exp Bot. 2022;73:5974–91 35436332 10.1093/jxb/erac165

[26] Wang YX, Liu XF, Sun M. et al. Melatonin enhances vase life and alters physiological responses in peony (*Paeonia lactiflora* Pall.) cut flowers. Postharvest Biol Technol. 2024;212:112896.

[27] Apel K, Hirt H. Reactive oxygen species: metabolism, oxidative stress, and signal transduction. Annu Rev Plant Biol. 2004;55:373–99 15377225 10.1146/annurev.arplant.55.031903.141701

[28] Liu D, Xu S, Hu H. et al. Endogenous hydrogen sulfide homeostasis is responsible for the alleviation of senescence of postharvest daylily flower via increasing antioxidant capacity and maintained energy status. J Agric Food Chem. 2017b;65:718–26 28060500 10.1021/acs.jafc.6b04389

[29] Wang Z, Liu X, Wang J. et al. Effect of flowering stages on the content of active ingredients and antioxidant capability of *Bletilla striata* flowers. Chem Biodivers. 2023;20:e202200773. 36629332 10.1002/cbdv.202200773

[30] Geng XM, Liu J, Lu JG. et al. Effects of cold storage and different pulsing treatments on postharvest quality of cut OT lily 'Mantissa' flowers. J Fac Agric - Kyushu Univ. 2009;54:41–5

[31] Shi J, Shi G, Tian Z. Effect of exogenous hydrogen peroxide or ascorbic acid on senescence in cut flowers of tree peony (*Paeonia suffruticosa* Andr.). J Hortic Sci Biotechnol. 2015;90:689–94

[32] Ma Z, Zhang K, Guo W. et al. Green synthesis of silver nanoparticles using *Eucommia ulmoides* leaf extract for inhibiting stem end bacteria in cut tree peony flowers. Front Plant Sci. 2023;14:1176359 37324696 10.3389/fpls.2023.1176359 PMC10266105

[33] Zhang Y, Xu YF, Shang WQ. et al. ClO_2_ treatment delays petal senescence and extends the vase life of *Paeonia suffruticosa* 'Luoyang Hong' cut flowers. Sci Hortic. 2024;325:112650.

[34] Song J, Li Y, Hu J. et al. Pre- and/or postharvest silicon application prolongs the vase life and enhances the quality of cut peony (*Paeonia lactiflora* Pall.) flowers. Plan Theory. 2021;10:1742 10.3390/plants10081742 PMC8398881 34451787

[35] Song J, Yang J, Jeong BR. Synergistic effects of silicon and preservative on promoting postharvest performance of cut flowers of peony (*Paeonia lactiflora* Pall.). Int J Mol Sci. 2022;23:13211 36362000 10.3390/ijms232113211 PMC9655603

[36] Zhao D, Cheng M, Tang W. et al. Nano-silver modifies the vase life of cut herbaceous peony (*Paeonia lactiflora* Pall.) flowers. Protoplasma. 2018;255:1001–13 29359232 10.1007/s00709-018-1209-1

[37] Li PY, Zhang WM, Tao J. et al. Herbaceous peony polyphenols extend the vase life of cut flowers. Agriculture. 2023b;13:122

[38] Guo YZ, Qiu YJ, Hu H. et al. Petal morphology is correlated with floral longevity in *Paeonia suffruticosa*. Agriculture. 2024;13:1372

[39] Bresson J, Bieker S, Riester L. et al. A guideline for leaf senescence analyses: from quantification to physiological and molecular investigations. J Exp Bot. 2018;69:769–86 28992225 10.1093/jxb/erx246

[40] Taiz L, Zeiger E. Plant Physiology. 5th ed. Sunderland, MA: Sinauer Associates; 2010:

[41] Domínguez F, Cejudo FJ. Chloroplast dismantling in leaf senescence. J Exp Bot. 2021;72:5905–18 33959761 10.1093/jxb/erab200 PMC8760853

[42] Shi GA . Physiological and Biochemical Mechanisms of Peony Flowering and Senescence (PhD dissertation). Huazhong: Huazhong Agricultural University; 2010: (in Chinese)

[43] Han CJ, Wang Q, Zhang HB. et al. Light shading improves the yield and quality of seed in oil-seed peony (*Paeonia ostii* Feng Dan). J Integrative Agric. 2018;17:1631–40

[44] Han C, Yu M, Wang Q. et al. Leaf structure and seed histochemistry analyses provided structural insights into the improved yield and quality of tree peony seed under light shading conditions. Sci Rep. 2020;10:4328 32152443 10.1038/s41598-020-61366-8 PMC7062827

[45] Zhao D, Luan Y, Shi W. et al. A *Paeonia ostii* caffeoyl-CoA *O*-methyltransferase confers drought stress tolerance by promoting lignin synthesis and ROS scavenging. Plant Sci. 2021;303:110765. 33487350 10.1016/j.plantsci.2020.110765

[46] Xie A, Lv M, Zhang D. et al. Effects of slight shading in summer on the leaf senescence and endogenous hormone and polyamine contents in herbaceous peony. Sci Rep. 2023;13:18714 37907675 10.1038/s41598-023-46192-y PMC10618196

[47] Wang Z, Shi GA, Ma XQ. et al. Ethylene metabolism and physiological mechanisms during the flowering and senescence of herbaceous peony 'Tao Hua Fei Xue'. Acta Hortic Sin (Nat Sci). 2014a;41:2268–74 (in Chinese)

[48] Han L . Study on the Mechanism of Low Temperature and Polyamines Affecting Postharvest Aging of Cut Peonies (MSc Thesis). Shandong: Shandong Agricultural University; 2016: (in Chinese)

[49] Rabiza-Świder J, Skutnik E, Jędrzejuk A. et al. Postharvest treatments improve quality of cut peony flowers. Agronomy. 2020;10:1583

[50] Xue JQ, Tang Y, Wang SL. et al. Assessment of vase quality and transcriptional regulation of sucrose transporter and invertase genes in cut peony (*Paeonia lactiflora* 'Yang Fei Chu Yu') treated by exogenous sucrose. Postharvest Biol Technol. 2018;143:92–101

[51] Sun J, Guo HX, Tao J. Effects of harvest stage, storage, and preservation technology on postharvest ornamental value of cut peony (*Paeonia lactiflora*) flowers. Agriculture. 2022;12:230

[52] Zhang C, Liu M, Fu JX. et al. Exogenous sugars involvement in senescence and ethylene production of tree peony 'Luoyang Hong' cut flowers. Korean J Hortic Sci Technol. 2013;30:718–24

[53] Wang W . Physiological Roles of ABA, H_2_O_2_, and Ethylene in the Senescence of Herbaceous Peony Cut Flowers. (MSc thesis). Henan: Henan University of Science and Technology; 2013: (in Chinese).

[54] van Doorn WG . Is petal senescence due to sugar starvation? Plant Physiol. 2004;134:35–42 14730063 10.1104/pp.103.033084 PMC1540351

[55] Skutnik E, Rabiza-Swider J, Jedrzejuk A. et al. The effect of the long-term cold storage and preservatives on senescence of cut herbaceous peony flowers. Agriculture. 2020;10:1631

[56] Zhang C, Wang Y, Fu J. et al. Transcriptomic analysis of cut tree peony with glucose supply using the RNA-Seq technique. Plant Cell Rep. 2014;33:111–29 24132406 10.1007/s00299-013-1516-0

[57] Xue JQ, Tang Y, Wang SL. et al. Evaluation of dry and wet storage on vase quality of cut peony based on the regulation of starch and sucrose metabolism. Postharvest Biol Technol. 2019;155:11–9

[58] Ma N, Ma C, Liu Y. et al. Petal senescence: a hormone view. J Exp Bot. 2018;69:719–32 29425359 10.1093/jxb/ery009

[59] Sun X, Qin M, Yu Q. et al. Molecular understanding of postharvest flower opening and senescence. Mol Hortic. 2021;1:7 37789453 10.1186/s43897-021-00015-8 PMC10514961

[60] Wang YJ, Zhang C, Jia PY. et al. Isolation and expression analysis of three EIN3-like genes in tree peony (*Paeonia suffruticosa*). Plant Cell Tissue Organ Cult. 2013b;112:181–90

[61] Wang YJ, Zhang C, Wang XQ. et al. Involvement of glucose in the regulation of ethylene biosynthesis and sensitivity in cut *Paeonia suffruticosa* flowers. Sci Hortic. 2014b;169:44–50

[62] Wu F, Zhang C, Wang XQ. et al. Ethylene-influenced development of tree peony cut flowers and characterization of genes involved in ethylene biosynthesis and perception. Postharvest Biol Technol. 2017;125:150–60

[63] Ji X, Wang M, Xu Z. et al. *PlMYB308* regulates flower senescence by modulating ethylene biosynthesis in herbaceous peony. Front Plant Sci. 2022;13:872442. 35712588 10.3389/fpls.2022.872442 PMC9194951

[64] Chen K, Li GJ, Bressan RA. et al. Abscisic acid dynamics, signaling, and functions in plants. J Integr Plant Biol. 2020;62:25–54 31850654 10.1111/jipb.12899

[65] Yuan YC, Zhou NN, Bai SS. et al. Evolutionary and integrative analysis of the gibberellin 20-oxidase, 3-oxidase, and 2-oxidase gene family in *Paeonia ostii*: insight into their roles in flower senescence. Agriculture. 2024;14:590

[66] Chandler JW . Auxin response factors. Plant Cell Environ. 2016;39:1014–28 26487015 10.1111/pce.12662

[67] Sun Y, Chen J, Yuan Y. et al. Auxin efflux carrier *PsPIN4* identified through genome-wide analysis as vital factor of petal abscission. Front Plant Sci. 2024a;15:1380417 38799094 10.3389/fpls.2024.1380417 PMC11116700

[68] Zhao DQ, Zhang XY, Wang R. et al. Herbaceous peony tryptophan decarboxylase confers drought and salt stresses tolerance. Env Exp Bot. 2019;162:345–56

[69] Cao JK . Cloning and Functional Analysis of the PlARF2 Gene in Herbaceous Peony (MSc thesis). Henan: Henan Agricultural University; 2023: (in Chinese)

[70] Zhou L, Zhang C, Fu JX. et al. Molecular characterization and expression of ethylene biosynthetic genes during cut flower development in tree peony (*Paeonia suffruticosa*) in response to ethylene and functional analysis of *PsACS1* in *Arabidopsis thaliana*. J Plant Growth Regul. 2013;32:362–375

[71] Liu CY, Liu ZQ, Yuan YC. et al. Comprehensive analyses of the proteome and ubiquitome revealed mechanism of high temperature accelerating petal abscission in tree peony. Hortic Plant J. 2024;10:205–22

[72] Tang Y, Zhang XX, Wang SL. et al. Effects of different cold storage methods on the vase quality of cut peonies. Northern Horticulture (Nat Sci). 2018;21:148–54 (in Chinese)

[73] Sun XH, Niu LX, Zhang MF. et al. Application of carbon-based nutrient fertilizer improved soil fertility and seed yield of *Paeonia ostii* 'Feng Dan'. Ind Crop Prod. 2024b;212:118348.

[74] Joshi K, Jugran AK, Bhatt I. Agrotechniques development for *Paeonia emodi* Royle: evaluation of soil composition, biomass, and secondary metabolites. J Soil Sci Plant Nutr. 2023;23:3290–301

[75] Hunter DA, Ferrante A, Vernieri P. et al. Role of abscisic acid in perianth senescence of daffodil (*Narcissus pseudonarcissus* "Dutch master"). Physiol Plant. 2004;121:313–21 15153199 10.1111/j.0031-9317.2004.0311.x

[76] do Nascimento Simões A, Diniz NB, da Silva Vieira MR. et al. Impact of GA_3_ and spermine on postharvest quality of anthurium cut flowers (*Anthurium andraeanum*) cv. Arizona. Sci Hortic. 2018;241:178–86

[77] Cheng C, Yu Q, Wang Y. et al. Ethylene-regulated asymmetric growth of the petal base promotes flower opening in rose (*Rosa hybrida*). Plant Cell. 2021;33:1229–51 33693903 10.1093/plcell/koab031

[78] Lu J, Zhang G, Ma C. et al. The F-box protein *RhSAF* destabilizes the gibberellic acid receptor *RhGID1* to mediate ethylene-induced petal senescence in rose. Plant Cell. 2024;36:1736–54 38315889 10.1093/plcell/koae035 PMC11062431

[79] Ma N, Xue J, Li Y. et al. *Rh-PIP2;1*, a rose aquaporin gene, is involved in ethylene-regulated petal expansion. Plant Physiol. 2008;148:894–907 18715962 10.1104/pp.108.120154 PMC2556823

[80] Eisinger W . Role of cytokinins in carnation flower senescence. Plant Physiol. 1997;59:707–9 10.1104/pp.59.4.707 PMC542477 16659922

[81] Macnish AJ, Jiang CZ, Reid MS. Treatment with thidiazuron improves opening and vase life of iris flowers. Postharvest Biol Technol. 2010;56:77–84

[82] Nagano S, Mori N, Tomari Y. et al. Effect of differences in light source environment on transcriptome of leaf lettuce (*Lactuca sativa* L.) to optimize cultivation conditions. PLoS One. 2022;17:e0265994. 35349601 10.1371/journal.pone.0265994 PMC8963549

[83] Shi J, Huang T, Zhang Y. et al. The effect of BVOCs produced by *Lysinibacillus fusiformis* and LED irradiation on pigment metabolism in stored broccoli. Food Chem. 2023;420:136068. 37043993 10.1016/j.foodchem.2023.136068

[84] Guan Y, Ding L, Jiang J. et al. Overexpression of the *CmJAZ1*-like gene delays flowering in *Chrysanthemum morifolium*. Hortic Res. 2021;8:87 33795661 10.1038/s41438-021-00525-y PMC8016864

[85] He S, Zhi F, Min Y. et al. 2023. The *MYB59* transcription factor negatively regulates salicylic acid- and jasmonic acid-mediated leaf senescence. Plant Physiol. 2023;192:488–503 36542529 10.1093/plphys/kiac589 PMC10152657

[86] Jiang L, Liu K, Zhang T. et al. The *RhWRKY33a-RhPLATZ9* regulatory module delays petal senescence by suppressing rapid reactive oxygen species accumulation in rose flowers. Plant J. 2023;114:1425–42 36951178 10.1111/tpj.16202

[87] Luo J, Chen S, Cao S. et al. Rose (*Rosa hybrida*) ethylene responsive factor 3 promotes rose flower senescence via direct activation of the abscisic acid synthesis-related *9-CIS-EPOXYCAROTENOID DIOXYGENASE* gene. Plant Cell Physiol. 2021;62:1030–43 34156085 10.1093/pcp/pcab085

[88] Xu H, Luo D, Zhang F. *DcWRKY75* promotes ethylene induced petal senescence in carnation (*Dianthus caryophyllus* L.). Plant J. 2021;108:1473–92 34587330 10.1111/tpj.15523

[89] Liu CY, Li FZ, Gai SP. et al. Screening and identification of genes associated with flower senescence in tree peony (*Paeonia* x *suffruticosa* Andrews) using suppression subtractive hybridization. J Hortic Sci Biotechnol. 2017a;92:146–54

[90] Xu J, Kang BC, Naing AH. et al. CRISPR/Cas9-mediated editing of 1-aminocyclopropane-1-carboxylate oxidase1 enhances *petunia* flower longevity. Plant Biotechnol J. 2020;18:287–97 31222853 10.1111/pbi.13197 PMC6920161

[91] Gautam T, Dutta M, Jaiswal V. et al. Emerging roles of *SWEET* sugar transporters in plant development and abiotic stress responses. Cells. 2022;11:1303 35455982 10.3390/cells11081303 PMC9031177

[92] Huo J, Huang D, Zhang J. et al. Comparative proteomic analysis during the involvement of nitric oxide in hydrogen gas-improved postharvest freshness in cut lilies. Int J Mol Sci. 2018;19:3955 30544843 10.3390/ijms19123955 PMC6320913

[93] Zhao H, Zhong S, Sang L. et al. *PaACL* silencing accelerates flower senescence and changes the proteome to maintain metabolic homeostasis in *Petunia hybrida*. J Exp Bot. 2020;71:4858–76 32364241 10.1093/jxb/eraa208 PMC7475263

[94] Corydon TJ, Schulz H, Richter P. et al. Current knowledge about the impact of microgravity on gene regulation. Cells. 2023;12:1043 37048115 10.3390/cells12071043 PMC10093652

[95] Cui X, Zheng J, Zheng J. et al. Study of autophagy in plant senescence. Methods Mol Biol. 2018;1744:299–306 29392674 10.1007/978-1-4939-7672-0_23

[96] Wang Y, Yu B, Zhao J. et al. Autophagy contributes to leaf starch degradation. Plant Cell. 2013a;25:1383–99 23564204 10.1105/tpc.112.108993 PMC3663275

[97] Li Y, Shu P, Xiang L. et al. CRISPR/Cas9-mediated SlATG5 mutagenesis reduces the resistance of tomato fruit to *Botrytis cinerea*. Food Secur. 2023a;12:2750 10.3390/foods12142750 PMC10380010 37509842

[98] Parmesan C, Hanley ME. Plants and climate change: complexities and surprises. Ann Bot. 2015;116:849–64 26555281 10.1093/aob/mcv169 PMC4640131

